# Packaged delivery of CRISPR–Cas9 ribonucleoproteins accelerates genome editing

**DOI:** 10.1093/nar/gkaf105

**Published:** 2025-02-27

**Authors:** Hannah Karp, Madeline Zoltek, Kevin Wasko, Angel Luis Vazquez, Jinna Brim, Wayne Ngo, Alanna Schepartz, Jennifer A Doudna

**Affiliations:** Department of Chemistry, University of California, Berkeley, CA 94720, United States; Innovative Genomics Institute, University of California, Berkeley, CA 94720, United States; Department of Molecular and Cell Biology, University of California, Berkeley, CA 94720, United States; Innovative Genomics Institute, University of California, Berkeley, CA 94720, United States; Department of Molecular and Cell Biology, University of California, Berkeley, CA 94720, United States; Department of Chemistry, University of California, Berkeley, CA 94720, United States; Department of Molecular and Cell Biology, University of California, Berkeley, CA 94720, United States; Innovative Genomics Institute, University of California, Berkeley, CA 94720, United States; California Institute for Quantitative Biosciences, University of California, Berkeley, CA 94720, United States; Gladstone Institute of Data Science and Biotechnology, Gladstone Institutes, San Francisco, CA 94158, United States; Department of Chemistry, University of California, Berkeley, CA 94720, United States; Department of Molecular and Cell Biology, University of California, Berkeley, CA 94720, United States; California Institute for Quantitative Biosciences, University of California, Berkeley, CA 94720, United States; ARC Institute, Palo Alto, CA 94304, United States; Chan Zuckerberg Biohub, San Francisco, CA 94158, United States; Department of Chemistry, University of California, Berkeley, CA 94720, United States; Innovative Genomics Institute, University of California, Berkeley, CA 94720, United States; Department of Molecular and Cell Biology, University of California, Berkeley, CA 94720, United States; California Institute for Quantitative Biosciences, University of California, Berkeley, CA 94720, United States; Gladstone Institute of Data Science and Biotechnology, Gladstone Institutes, San Francisco, CA 94158, United States; Howard Hughes Medical Institute, University of California, Berkeley, CA 94720, United States

## Abstract

Effective genome editing requires a sufficient dose of CRISPR–Cas9 ribonucleoproteins (RNPs) to enter the target cell while minimizing immune responses, off-target editing, and cytotoxicity. Clinical use of Cas9 RNPs currently entails electroporation into cells *ex vivo*, but no systematic comparison of this method to packaged RNP delivery has been made. Here we compared two delivery strategies, electroporation and enveloped delivery vehicles (EDVs), to investigate the Cas9 dosage requirements for genome editing. Using fluorescence correlation spectroscopy, we determined that >1300 Cas9 RNPs per nucleus are typically required for productive genome editing. EDV-mediated editing was >30-fold more efficient than electroporation, and editing occurs at least 2-fold faster for EDV delivery at comparable total Cas9 RNP doses. We hypothesize that differences in efficacy between these methods result in part from the increased duration of RNP nuclear residence resulting from EDV delivery. Our results directly compare RNP delivery strategies, showing that packaged delivery could dramatically reduce the amount of CRISPR–Cas9 RNPs required for experimental or clinical genome editing.

## Graphical Abstract

**Graphical Abstract:**
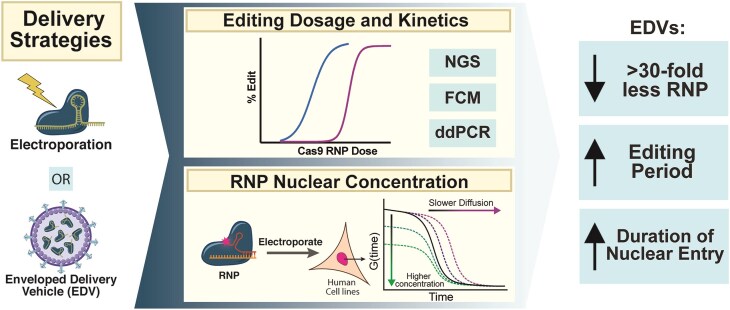

## Introduction

CRISPR-based genome editing therapies have enormous potential to cure genetic diseases. Despite this promise, safe and effective delivery of genome editors remains a challenge for both therapeutic development and fundamental research [1,2]. Broadly speaking, genome editors can be delivered either as a nucleic acid, to be transcribed and/or translated in the target cell, or as an intact ribonucleoprotein (RNP) complex [1]. There are distinct advantages to delivering genome editors as RNPs, including shorter intracellular lifetimes that minimize off-target edits [3–5] and reduce immunogenicity [6–8]. Compared to messenger RNA (mRNA) delivery, RNP delivery may result in lower levels of toll-like receptor activation [6, 9] and enable higher *in vivo* editing efficacy by bypassing *in situ* translation of mRNA [10] and protecting the guide RNA (gRNA) integrity due to Cas9 protein binding [5]. RNP delivery also avoids risks of random DNA integration posed by viral vectors, including lentivirus and adeno-associated virus [11,12].

While delivery of proteins and RNA to the interior of cells remains a critical therapeutic challenge [13–15], extensive engineering efforts have generated multiple promising *ex vivo* and *in vivo* Cas9 RNP delivery strategies [1,2]. RNP electroporation is the most common strategy, currently used in several CRISPR genome editing therapies for blood disorders [16,17]. RNP electroporation is less cytotoxic than nucleic acid electroporation [18], is efficient in primary cells, and has higher specificity than delivery systems that result in extended genome editor expression [3]. However, RNP electroporation requires an *ex vivo* approach, limiting its therapeutic utility. Furthermore, electroporation can impact cell viability [19], which increases the overall costs and production time frame for cell-based therapies [19]. Alternatively, enveloped delivery vehicles (EDVs), derived from retrovirus, offer a packaged approach to Cas9 RNP delivery, which safeguards cell integrity by utilizing endogenous endocytic uptake for cell entry [20–25]. EDVs leverage intrinsic viral intracellular delivery capabilities while mitigating the risk of lentiviral genome integration or extended transgene expression [20, 26, 27]. EDVs use vesicular stomatitis virus glycoprotein, VSVG, for cellular uptake and endosomal escape, which typically exhibits broad cell tropism [12, 20, 21, 26, 28]. Recent work demonstrated that binding-deficient VSVG combined with antibody-derived targeting motifs enables cell-type-specific Cas9 delivery both *ex vivo* and *in vivo* [26, 29]. However, despite the promise of RNPs and EDVs, little is known about how much functional Cas9 RNP can be delivered in each case and how much is required for efficient editing in human cells [3, 8, 19, 30].

To address these questions, we compared electroporation and EDVs for the delivery of *Streptococcus pyogenes* Cas9 RNPs to edit various human cell types. We determined the impact of delivery modality on the rate of DNA cleavage and repair. Using fluorescence correlation spectroscopy (FCS), we found that >1300 Cas9 RNPs per nucleus are required for editing in human cell lines. At comparable Cas9 RNP doses, EDVs are 30- to 50-fold more effective at editing, across multiple human cell types and target genome sequences. Furthermore, EDV delivery generates genome edits twice as fast as electroporation. We hypothesize that the observed differences in editing efficacy and rate result in part from differences in RNP versus EDV trafficking to the cell nucleus. Our results suggest that the Cas9 RNP dosage used for current *ex vivo* research and clinical genome editing could be substantially reduced by switching from electroporation to a packaged delivery strategy such as EDVs. These findings also reveal the importance of delivery modality for genome editing efficacy and pave the way for engineering optimal delivery methods to ensure maximal genome editing with minimal side effects.

## Materials and methods

### Plasmid construction

Restriction enzymes used in this study were purchased from New England Biolabs (NEB). Plasmids were constructed using NEBuilder HiFi DNA Assembly Master Mix (NEB) with polymerase chain reaction (PCR) products and backbone restriction digests. For guide plasmid cloning, protospacer oligos were annealed and then inserted using BsmBI golden gate assembly into the optimized Gag-Cas9 (Gag-3xNES-2xNLS-Cas9-U6-sgRNA) and PsPax-U6-sgRNA plasmids as previously reported [26]. Oligos encoding the single-guide RNA (sgRNA) spacers (IDT), and all other oligos used in this study, are outlined in Supplementary Table S3.

Cloning and DNA preparations were performed in MultiShot StripWell Mach1 (Thermo Fisher). All plasmids used in tissue culture were prepared with Qiagen Plasmid Maxi Kit (Qiagen). All plasmids were sequence confirmed prior to use (Plasmidsaurus, UC Berkeley DNA Sequencing Facility).

### Tissue culture

Lenti-X (Takara Biosciences), HEK293T, U2OS, and HeLa cells were obtained and authenticated by the UC Berkeley Cell Culture Facility. Lenti-X, HEK293T, U2OS, and HeLa cell lines were cultured in Dulbecco’s modified Eagle medium (DMEM; Fisher Scientific) supplemented with 100 U/ml penicillin–streptomycin (Thermo Fisher) and 10% (v/v) fetal bovine serum and passaged with trypsin–EDTA (0.25%, Phenol Red, Fisher Scientific).

### RNP electroporation

sgRNA (IDT, Supplementary Table S1) was resuspended in IDT duplex buffer to 100 μM concentration. Cas9 RNPs were formed by combining the sgRNA and 40 μM Cas9-NLS (UC Berkeley QB3 MacroLab) at a molar ratio of 1.5:1 and incubating at room temperature for 10–15 min. Electroporation was performed using a 96-well format 4D-nucleofector (Lonza) with 10^5^ cells per well (unless otherwise specified). HEK293T cells were electroporated with the SF buffer and the CM-130 pulse code. HeLa cells were electroporated with SE buffer and the CN-114 pulse code. For cell lines, cells were immediately resuspended in prewarmed media and transferred to culture plates.

### EDV and lentiviral production

Cas9-EDVs were produced as previously described [20, 26]. Briefly, Cas9-EDVs were produced by seeding ∼4 million Lenti-X cells (Takara Bio) into 10-cm tissue culture dishes (Corning) and transfecting the next day with 1 μg pCMV-VSV-G (Addgene plasmid #8454), 6.7 μg Gag-Cas9-U6-sgRNA, and 3.3 μg psPax2-U6-sgRNA (Addgene plasmid #12260) using TransIT-LT1 (Mirus Bio) at a 3:1 TransIT-LT1:plasmid ratio. Two days post-transfection, Cas9-EDV-containing supernatants were harvested, passed through a 0.45-μm PES syringe filter (VWR), and concentrated with ultracentrifugation by laying EDV-containing supernatant on top of 30% sucrose in 100 mM NaCl, 10 mM Tris–HCl (pH 7.5), and 1 mM EDTA at 25 000 rpm with an SW28 rotor (Beckman Coulter) for 2 h at 4°C in polypropylene tubes (Beckman Coulter). Concentrated Cas9-EDVs were resuspended in Opti-MEM (Gibco) at a final concentration of 20× unless otherwise noted and frozen at −80°C until use.

### Fluorescence correlation spectroscopy

On the day of each FCS experiment, 300 μl of 10–100 nM Alexa Fluor 594 hydrazide for electroporation experiments using ATTO™ 550 for Cas9 detection, both diluted into Milli-Q, was added to one well of the eight-well microscopy dish and incubated at 37°C for at least 30 min. Immediately prior to measurements, a DNA stain, 300 nM Hoechst 33342, was incubated with the cells for 5 min to visualize nuclei. After nuclear dye incubation, cells were washed 2× with Dulbecco’s phosphate-buffered saline (DPBS) and incubated with prewarmed DMEM for imaging.

The general procedures used for FCS have been described previously [31–33]. Experiments were performed with a STELLARIS 8 microscope (Leica Microsystems) with a Leica DMi8 CS scanhead, an HC Plan-Apo 63×/1.4NA water immersion objective, and a pulsed white-light laser (440–790 nm; 440 nm: >1.1 mW; 488 nm: >1.6 mW; 560 nm: >2.0 mW; 630 nm: >2.6 mW; 790 nm: >3.5 mW, 78 MHz). All confocal imaging was performed using HyD S or HyD X detectors in counting mode, while FCS measurements were carried out using only a Hybrid HyD X detector in counting mode. All microscopy experiments were performed at 37°C (monitored using Oko-Touch) and 5% CO_2_ in a blacked out cage enclosure (Okolab). Before each experiment, the correction collar of the objective was adjusted by maximizing the counts per molecule for the Alexa Fluor 594 hydrazide dye standard; minor fluctuations in the correction collar are expected based on the variable thickness of the glass-bottom microscopy dishes (LabTek™). After correction collar adjustment, ten 5-s autocorrelation traces were obtained from the well containing dye standard to calculate the focal volume of the microscope (see the “Analysis of FCS data” section for more details). Alexa Fluor 594 was measured using the same settings as ATTO™ 550.

For electroporation experiments, ATTO™ 550 was excited at 553 nm with an emission window of 570–660 nm, and Hoechst 33342 was excited at 405 nm with an emission window of 432–509 nm. Laser intensity for ATTO™ 550 was determined using *in vitro* samples of each respective protein and guide and determining the maximal laser intensity for which the observed counts per molecule remained within a linear range. The pinhole S5 of the laser was set to 1 au. A confocal microscopy image of the cells was used to position the crosshairs of the microscope laser in the nucleus of 10–15 cells within the frame. All FCS measurements consisted of ten 10-s traces. A minimum of 30 cells per condition were measured for each biological replicate, and a minimum of two biological replicates were collected for each condition.

The expected diffusion time (*τ*_diff_) for ATTO™ 550-Cas9 RNP was obtained by measuring *in vitro* autocorrelation traces for 400 nM solutions of RNP, annealed ATTO™ 550-tracrRNA:crRNA, or ATTO™ 550-tracrRNA alone in DMEM media (25 mM HEPES, no Phenol Red) at 37°C (Supplementary Fig. S6). For each sample, ten 10-s autocorrelation traces using the settings described earlier were measured per point, and three points were obtained per sample. Given the distribution of *τ*_diff_ values measured for free RNA in buffer versus in cells, we employed a lower *τ*_diff_ filter of 0.5 ms for Cas9 RNP delivery experiments to avoid fluorescent signal contributed by free RNA. These data were fitted using Equation (1) to derive the average diffusion time (*τ*_diff_).

### Analysis of FCS data

Autocorrelation traces obtained from FCS measurements were analyzed using a custom MATLAB script [32,33]. To extract quantitative information from *in cellulo* data, the effective confocal volume of the microscope must be known. This value was determined using Equations (1)–(3) and by the *in vitro* autocorrelation traces for the Alexa Fluor 594 hydrazide standard measured at the start of each experiment, which have known diffusion coefficients in water [34,35]. These traces were fitted to a 3D diffusion equation:


(1) \begin{eqnarray*}
G\left( \tau \right) = \frac{1}{N} \cdot \frac{1}{{\left( {1 + \frac{\tau }{{\tau_{\rm diff}}}} \right)\sqrt {1 + \left( {{s^2}\frac{\tau }{{\tau_{\rm diff}}}} \right)} }},
\end{eqnarray*}

where *N* is the average number of molecules detected in the focal volume (*V*_eff_), *τ*_diff_ is the average diffusion time that a molecule requires to cross *V*_eff_, and *s* is the structure factor (the ratio of the radial to axial dimensions of the focal volume). The structure factor was measured to be 0.17 using the autocorrelation function of Alexa Fluor 594 in water at 25°C and fixed for all subsequent analysis. *V*_eff_ can be extracted from these data by inserting the *τ*_diff_ value derived from Equation (1) to calculate *ω*_1_ in Equation (2):


(2) \begin{eqnarray*}
{\omega _1} = \sqrt {4 \cdot D \cdot {\tau _{{\rm diff}}}},
\end{eqnarray*}

where *ω*_1_ is the lateral extension of the confocal volume and *D* is the known diffusion coefficient of the dye standard in water at 37°C. Note that diffusion coefficients at 25°C are typically reported in the literature and can be used to calculate the diffusion coefficient at 37°C using Equation (3):


(3) \begin{eqnarray*}
D\left( T \right) = D\left( {25^\circ {\rm C}} \right) \cdot \;\frac{{t + 273.15}}{{\eta \left( t \right)}}\; \cdot 2.985 \times {10^{ - 6}}\;{\rm Pa}\; {\rm s} /{{\rm K}} ,\nonumber\\
\end{eqnarray*}

where *t* = 37°C, *D*(25°C) for Alexa Fluor 594 is 3.88 × 10^−6^ cm^2^/s and for Alexa Fluor 488 is 4.14 × 10^−6^ cm^2^/s [36], and *η*(*t*) is the viscosity of water at 37°C (6.913 × 10^−4^ Pa s) [30]. Using this formula, the diffusion coefficient of Alexa Fluor 594 at 37°C is 5.20 × 10^−6^ cm^2^/s and that of Alexa Fluor 488 is 5.54 × 10^−6^ cm^2^/s.

*V*_eff_ can then be directly calculated from the following equation:


(4) \begin{eqnarray*}
{V_{{\rm eff}}} = {\pi ^{{3}/{2}}} \cdot \left( {{\omega _1}^3} \right) \cdot \frac{1}{s}.
\end{eqnarray*}

The average *V*_eff_ for all experiments ranged from 0.25 to 0.4 fl.

To determine the appropriate fitting equation for data collected in cells, autocorrelation traces derived from *in cellulo* measurements of HeLa cells treated with 0.24 × 10^7^ to 15 × 10^7^ Cas9 per cell were fitted using two equations. The first was a 3D anomalous diffusion equation used for previous FCS measurements of proteins in live cells [31]:


(5) \begin{eqnarray*}
G\left( \tau \right) = \frac{1}{N} \cdot \frac{1}{{{{\left( {1 + \frac{\tau }{{\tau_{\rm diff}}}} \right)}^\alpha }{{\sqrt {1 + {s^2}\left( {\frac{\tau }{{{\tau _{{\rm diff}}}}}} \right)} }^\alpha }}} + G\left( \infty \right),
\end{eqnarray*}

where *N* is the average number of molecules in the focal volume, *τ*_diff_ is the average diffusion time that a molecule requires to cross *V*_eff_, *α* is the anomalous diffusion coefficient, and *s* is the structure factor (0.17).

The second equation was a two-component diffusion equation previously applied to analysis of DNA-binding transcription factors by FCS [33,37]. The equation incorporates both a rapidly and a slowly diffusing component to account for biphasic autocorrelation functions. This equation is almost identical to a two-component diffusion equation used for previous single-molecule analysis of Cas9 in live cells [38], except it incorporates an anomalous diffusion coefficient for the slow-diffusing fraction [33]. Since Cas9 binds DNA, we expected Equation (6) to more accurately fit autocorrelation functions in live cells than Equation (5):


(6) \begin{eqnarray*}
G\left( \tau \right) &=& \frac{1}{N}\left( {{F_{{\rm fast}}} \cdot \frac{1}{{\left( {1 + \frac{\tau }{{{\tau _{{\rm diff}1}}}}} \right) \cdot \sqrt {1 + {s^2}\left( {\frac{\tau }{{{\tau _{{\rm diff}1}}}}} \right)} }}} \right)\nonumber\\ && \times \left( {\left( {1 - {F_{{\rm fast}}}} \right) \cdot \frac{1}{{(1 + {{(\frac{\tau }{{{\tau _{{\rm diff}2}}}})}^\alpha }\sqrt {1 + {s^2}{{\left( {\frac{\tau }{{{\tau _{{\rm diff}2}}}}} \right)}^\alpha }} }}} \right),\nonumber\\
\end{eqnarray*}

where *N* is the average number of molecules in the focal volume, *τ*_diff1_ is the average diffusion time for the rapidly diffusing component, *τ*_diff2_is the average diffusion time for the slow-diffusing component, *α* is the anomalous diffusion coefficient, *F*_fast_ is the fraction of molecules that are rapidly diffusing, and *s* is the structure factor (0.17).

From the fittings, a set of parameters specific to individual measurements was obtained, including the diffusion time of the detected molecules (a single *τ*_diff_ for the one-component fit or *τ*_diff1_ and *τ*_diff2_ for the two-component fit), the fraction of molecules rapidly diffusing (*F*_fast_), the number of molecules detected in the focal volume, and a chi-square (*χ*^2^) value to describe the goodness of fit. The *χ*^2^ values were compared for the one-component versus two-component fits to determine that the two-component diffusion equation most accurately represents the data. All FCS data were therefore fitted by Equation (6) and filtered as described previously [33] to remove measurements where *τ*_diff1_< 0.5 ms (indicative of free RNA), *τ*_diff1_> 10 ms (indicative of aggregation), *α* < 0.3, and *χ*^2^ > 30. We also excluded fits for which a second component was not identified.

For all FCS curves that passed these thresholds, the concentration (*C*) of protein in the nucleus was then calculated using the value of *N* derived earlier in Equation (7):


(7) \begin{eqnarray*}
C = \frac{N}{{{N_{\rm A}} \cdot {V_{{\rm eff}}}}},
\end{eqnarray*}

where *N*_A_ is Avogadro’s number (6.023 × 10^23^ mol^−1^). At least 20 concentration values from curves that passed all filters were used for each FCS condition.

The number of Cas9 molecules in the nucleus was calculated using the concentration obtained from Equation (7) and a HeLa nuclear volume of 6.90 × 10^−13^ l [32] using Equation (8):


(8) \begin{eqnarray*}
{N_{{\rm nucleus}}} = C \cdot \left( {6.9 \times {{10}^{ - 13}}\;{\rm l}} \right) \cdot \;{N_{\rm A}},
\end{eqnarray*}

where *N*_A_ is Avogadro’s number (6.023 × 10^23^ mol^−1^).

Finally, each concentration value derived from Equation (7) had a corresponding *F*_fast_ value describing the fraction of this concentration that was rapidly diffusing. The concentration of DNA-bound Cas9 in the nucleus was then calculated using Equation (9):


(9) \begin{eqnarray*}
{C_{{\rm bound}}} = \frac{1}{{{F_{{\rm fast}}}}}\; \cdot C.
\end{eqnarray*}

### Cas9 molecules per nucleus calculations

The number of Cas9 molecules per nucleus was calculated by multiplying the average volume of the HeLa nuclei, 690 μm^3^ [39], by the nuclear concentration determined by FCS. To estimate the number of Cas9 RNPs per nucleus that are typically required for productive editing by nucleofection, we first estimated the total Cas9 doses required for editing by electroporation by analyzing the HeLa dose curves (Supplementary Fig. S3B). The median Cas9 RNP dosage for half maximal (EC50) editing by electroporation was 6.4 × 10^7^ Cas9 per cell (Supplementary Fig. S3B). The high-efficiency B2M guide (Supplementary Table S3) EC50 was 7.2 × 10^6^ Cas9 per cell in HeLa cells. Using the linear regression from the FCS electroporation dose titration (Fig. 2D and Supplementary Table S2), we calculated that this dosage would amount to nuclear concentration of 3.2 nM. Using Avogadro’s number (6.023 × 10^23^ mol^−1^), this amounts to ∼1300 Cas9 RNP molecules per nucleus.

### Western blotting and densitometry

Samples were denatured by mixing with 5× Laemmli with 10% 2-mercaptoethanol and heating at 95°C for 3 min. Samples were run on 4%–20% SDS–PAGE (sodium dodecyl sulfate–polyacrylamide gel electrophoresis) gels (Bio-Rad) prior to transfer onto a methanol-soaked polyvinylidene difluoride (PVDF, Bio-Rad) membrane. PVDF membranes were blocked with 10% non-fat milk (Apex) in 1× PBS (Gibco) with 0.1% Tween (Sigma) (PBS-T) for 1 h at room temperature (∼22–25°C). The solution was replaced with 0.1% non-fat milk in PBS-T and primary antibody dilution (Supplementary Table S1) in 1% non-fat milk in PBS-T incubated at 4°C overnight. The following day, the solution was replaced with 1% non-fat milk in PBS-T and a secondary antibody dilution (Supplementary Table S1) and gently shaken for 1 h. Western blot membranes were washed with PBS-T three times, with 2–3-min wash steps, prior to imaging on a LI-COR OdysseyCLx. Fiji (previously ImageJ) was used to quantify relative band intensity on western blots.

### Quantification of Cas9 RNPs per EDV

The Cas9 ELISA Kit (Cell Biolabs Inc.) and Lenti-X p24 Rapid Titer Kit (Takara Biosciences) were used to quantify the cas9 and p24 in Cas9-EDVs, respectively. For cas9 and p24 measurements, Cas9-EDVs were diluted 20–2000-fold and 1000–100 000-fold, respectively. Both ELISAs were done according to the manufacturer’s protocol. Absorbance at 450 nm was measured by a plate reader (Biotek). The amount of Cas9 in the samples was determined by comparison to serial dilution to a cas9 standard (Cell Biolabs Inc.). The amount of p24 was determined by comparison to serial dilution to a p24 standard (Takara Biosciences). The number of p24 per EDV was approximated to be 2500 CA molecules per particle [40].

Cas9 from lysed EDVs were measured by ELISA and validated against purified Cas9 from commercial (IDT, Cell Biolabs) and in-house sources (UC Berkeley QB3 MacroLab), quantified with photospectrometry (Supplementary Fig. S2).

### Quantitative RT-PCR of B2M sgRNA in EDVs

Quantitative RT-PCR was done as previously published [26]. Cas9-EDVs containing the sgRNA targeting the B2M gene were produced and concentrated as described earlier. RNA was extracted from 150 μl of Cas9-EDVs using the NucleoSpin RNA Virus Kit (Takara Bio) following the manufacturer’s instructions. Quantitative RT-PCR was done with PrimeTime™ One-Step RT-qPCR Master Mix (IDT) following the manufacturer’s instructions for the QuantStudio 6 Flex Real-Time PCR System (Thermo Fisher). The qPCR primers were custom ordered as a TaqMan Small RNA Assay to detect the B2M sgRNA (Supplementary Table S3)

### *In vitro* cleavage assessment of Cas9 activity

Prior to use, double-stranded DNA (dsDNA) substrate (Supplementary Table S1) was annealed in 10 mM HEPES, 20 mM KCl, and 1.5 mM MgCl_2_ at 95°C for 5 min and cooled at a rate of 1°C/min to 4°C. The annealed substrate was run on 8% native PAGE and annealed band was excised, ground finely, and eluted in 5 ml of water overnight at 4°C. Next day, this solution was filtered through a 0.22-μm filter, concentrated with 3-kDa spin filter (Amicon), and ethanol precipitated. The dried pellet was resuspended in diethyl pyrocarbonate (DEPC) treated water, and the concentration was determined by spectrophotometry.

Single guide targeting the spacer was complexed with Cas9 protein in 2:1 ratio for 15 min at 37°C in 200 mM HEPES, 1 M KCl, 100 mM MgCl_2_, 10% glycerol, and 5 mM dithiothreitol (DTT). Following complexation, the RNP was diluted and added in 1:1 stoichiometry to annealed dsDNA substrate (Supplementary Table S1) at final concentrations of 100 nM. The cleavage reaction occurred at 37°C for 2 h in 20 mM HEPES, 100 mM KCl, 10 mM MgCl_2_, 1% glycerol, and 0.5 mM DTT.

### Next-generation sequencing to assess genome editing

Next-generation sequencing (NGS) was used for detection of on-target genome editing in 293T cells. Genomic DNA was extracted using QuickExtract (Lucigen) as previously described [20]. Q5 high-fidelity polymerase (NEB) was used to attach adapters to the Cas9-RNP target site amplicons (Supplementary Table S1). The resulting PCR1 products were cleaned up using magnetic SPRI beads (UC Berkeley DNA Sequencing Facility). Library preparation and sequencing was performed by the Innovative Genomics Institute Next Generation Sequencing Core using MiSeq v2 (Illumina). Reads were analyzed with CRISPResso2 (http://crispresso.pinellolab.partners.org/login).

### Digital droplet quantitative PCR

Cells were collected at day 4, unless otherwise stated in figures, following editing with electroporation or EDVs, and genomic DNA was extracted with QuickExtract DNA Extraction Solution (Lucigen).

For double-strand DNA break (DSB) detection, the digital droplet quantitative PCR (ddPCR) setup was similar to what has been previously described [41,42], with two ∼200-bp amplicons for the *B2M* target gene (Supplementary Table S3). Amplicon 1 was located proximal to the centromere and utilized a 5′-hexachlorofluorescein (HEX)-labeled oligonucleotide probe (PrimeTime qPCR probes, Zen double quencher, IDT). Amplicon 2 was located ∼200 bp away from amplicon 1 and utilized a 5′,6-fluorescein (FAM)-labeled oligonucleotide probe (PrimeTime qPCR probes, Zen double quencher, IDT). Amplicon 1 served as a reference that should be unaffected by Cas9 genome editing and would signal whether *B2M* was in a given droplet. Amplicon 2 spanned the *B2M* target site, with the probe located ∼50 bp from the cleavage site. If the target site was not repaired after Cas9 cleavage, or if the chromosome was lost [41], amplicon 2 would not be amplified and the FAM probe would be quenched. ddPCR reactions were assembled with ddPCR Supermix for Probes (No dUTP, Bio-Rad), 900 nM of each primer, 250 nM of each probe, and 15–30 ng gDNA.

Droplets were formed using a QX200 Droplet Generator (Bio-Rad) following the manufacturer’s instructions prior to PCR. The next day, ddPCR droplets were analyzed on a QX200 Droplet Reader (Bio-Rad). Data were analyzed with the QX Manager Software (Bio-Rad), and thresholds were set manually based on wells with untreated reference samples. The percentage of DSBs was calculated based on droplets that had the reference amplicon 1 (HEX+) but did not produce the neighboring amplicon (FAM+):


\begin{eqnarray*}
\% {\rm DSB} = 100\times \{ {1 - ( {[ {{\rm FAM}} ]/[ {{\rm HEX}} ]} ) \}} .
\end{eqnarray*}

### Immunofluorescent imaging and quantification

For imaging EDV delivery in HeLa cells, 400 000 cells were plated the evening prior in a 12-cm dish that had 18-mm coverslips precoated with poly-l-lysine (Sigma–Aldrich, #P7886, 100 μg/ml in 75 mM NaCl, 50 mM Na_2_H_20_B_4_O_17_, pH 8.4). For EDV experiments, unless otherwise stated in figure legends, 350 μl of 20× concentrated EDV mixture was added to the cells in 1:1 mix with Opti-MEM (final volume 700 μl). EDV-containing medium was swapped for prewarmed supplemented DMEM (see earlier) at ∼4 h. For imaging Cas9 delivered by electroporation, 3 million cells were electroporated with 600 pmol RNP (1.2 × 10^8^ Cas9 per cell). Following delivery, at time points specified in the figures, cells were fixed with 4% paraformaldehyde in 1× DPBS for 10 min (Thermo Scientific, #28908). For time course experiments, all cells were fixed and then stored in 1× DPBS until all samples were harvested, so that permeabilization and staining steps were done simultaneously. We then washed coverslips three times with 1× DPBS and permeabilized samples with 0.2% Triton X-100 in 1× DPBS for 10 min. Following permeabilization, samples were washed three times and then blocked with Image-iT FX signal enhancer (Invitrogen). After this initial blocking step, samples were washed twice with 1× DPBS and then further blocked in 10% goat serum (Thermo Fisher, #50062Z) for 20–30 min. Then, samples were incubated with primary antibodies (Supplementary Table S1) diluted in 10% goat serum at 4°C overnight. The following day, the coverslips were washed three times with 1× DPBS and incubated for 1 h with secondary antibodies (Supplementary Table S3). For EDV confocal microscopy, secondary antibodies from tyramide signal superboost kits (Thermo Fisher; Supplementary Table S3) were used following manufacturer’s instructions. The labeling reaction was done for 2 min for all EDV experiments. Samples were then washed three times and mounted with ProLong Diamond antifade mountant (Invitrogen). All slides were stored at −20°C for periods longer than 1 week. All fixed cell confocal microscopy was performed using the Zeiss LSM710 microscope (UC Berkeley Bioimaging Facility).

For quantification of relative nuclear intensity of Cas9 and colocalization analysis, all *Z*-stacks were obtained based on half the wavelength of emission of the Cas9-associated fluorophore (Nyquist sampling) through the *Z*-plane of the HeLa cells that were counterstained with SYTOX™ Deep Red Nucleic Acid Stain (1:2000, Invitrogen, #S11380). These images were deconvoluted using the default settings of Huygens Professional (v22.10) to reduce noise. For the colocalization analysis, these images were thresholded using the Costes method [43] and for each image a Pearson’s correlation was calculated. For the relative Cas9 nuclear intensity quantification, the entire nuclear counterstained region was outlined and the median nuclear intensity in the Cas9 channel was quantified using Imaris (v10.2). Any nuclei that were on the edges of the image, were actively dividing, or could not be independently quantified due to their proximity to other nuclei were manually excluded from quantification.

### Flow cytometry

Cells were stained with anti-human B2M-PE (316306, BioLegend) in PBS containing 1% bovine serum albumin. An Attune NxT flow cytometer equipped with a 96-well autosampler (Thermo Fisher Scientific) was used for flow cytometry acquisition. Data analysis was performed using FlowJo v10.7.1 (FlowJo, LLC, Ashland, OR).

### Statistical analysis

Statistical analysis was performed using Prism v10, unless otherwise stated. Statistical details for experiments, including the values and definitions of the sample sizes and error bars, are reported in the figure legends. Unless otherwise specified in figure legends, two sided *t*-tests were used for pairwise comparisons and ANOVA (analysis of variance) was used for multiple comparisons.

## Results

### Several thousand Cas9 RNPs per cell nucleus are sufficient for editing human cell lines

**Figure 1.**
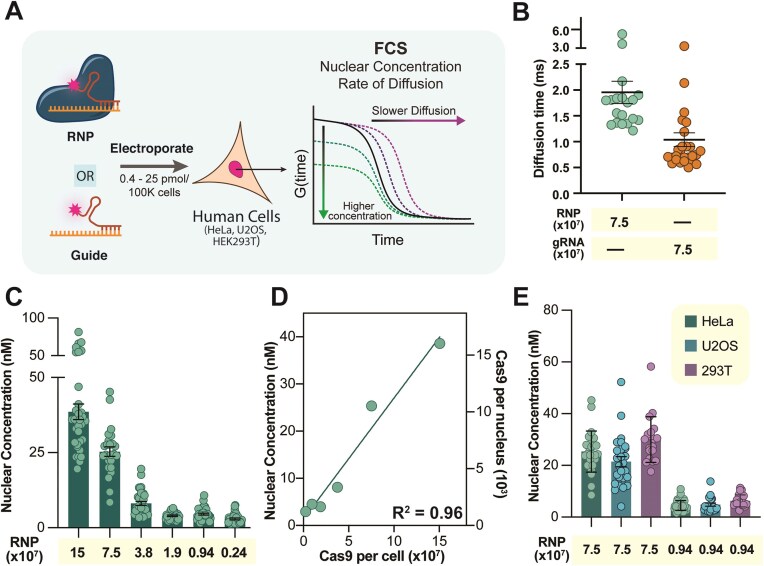
Quantifying Cas9 RNP nuclear concentration delivered by electroporation with FCS. (**A**) Experimental schematic of workflow to quantify the Cas9 RNP nuclear concentration required for editing. (**B**) Diffusion time of Cas9 RNP or gRNA, in Cas9 per cell, delivered in HeLa cells and measured at 24 h (2.0 ms versus 1.0 ms, *P*-value = .0004) Each point represents the average diffusion time in an individual cell modeled with a two-component diffusion fitting (Supplementary Fig. S1). FCS diffusion times are provided in ms; *n* > 25 for each FCS condition with at least two biological replicates each [mean ± standard error of the mean (SEM)]. (**C**) FCS analysis of HeLa cells electroporated with the Cas9 RNP. Nuclear concentration of Cas9 RNP as a function of dosage (in Cas9 per cell). Each point represents the concentration in an individual cell. FCS values are provided in nM; *n* > 25 for each FCS condition with at least two biological replicates each (mean ± SEM). All concentration values and diffusion times were derived by fitting FCS traces with a two-component 3D diffusion equation (see the “Materials and methods” section for more details). (**D**) (Left axis) Average nuclear concentration of Cas9 RNP versus dosage shows a strong linear correlation (*R*^2^ = 0.96). (Right axis) Estimated number of Cas9 per nucleus calculated from nuclear concentration values measured by FCS and volume of HeLa nucleus (690 μm^3^) [39]. (**E**) FCS analysis of nuclear concentration for HeLa, U2OS, and HEK293T cells. FCS values are provided in nM; *n* > 20 for each FCS condition with at least two biological replicates each (mean ± SEM). Exact values for FCS, including experimental and biological replicates, mean, and SEM, are reported in Supplementary Table S2.

To estimate the number of Cas9 RNP molecules per cell nucleus that are sufficient for editing, we used FCS to measure both the concentration and rate of diffusion of fluorescently labeled RNPs in cells (Fig. 1A) [44–46]. Briefly, FCS measures fluctuations of fluorescence emissions over time for a population of single molecules within a known focal volume [31, 34, 37]. These time-dependent fluctuations are fit to an autocorrelation function that provides quantitative information about the diffusion rate and concentration of molecules within the cellular environment [31, 32, 37,45, 47, 48]. We incubated purified Cas9 protein with a dual-guide RNA consisting of commercially available ATTO™ 550-labeled *trans*-activating CRISPR RNA (tracrRNA) and a *B2M*-targeting CRISPR RNA (crRNA) (Supplementary Table S1). For RNP-based applications, previous research has found minimal differences between dual guides, composed of separate tracrRNA and crRNA, and fused single guides (sgRNA), and these were used interchangeably [49–51]. As we were most interested in quantifying the amount of intact RNP, we reasoned that fluorescently labeled gRNA would provide a detectable shift in diffusion time between free RNA and the intact RNP in buffer (Supplementary Fig. S1A and B) and in cells. We compared the concentration and diffusion rates of electroporated Cas9 pre-complexed with the labeled dual-guide RNA versus the labeled dual-guide RNA alone in HeLa cells (Fig. 1A). FCS requires that cells be fully adherent and immobile [31,44–46]. After 24 h, to allow electroporated cells to recover and re-adhere, fluorescent signal in nuclei was evaluated by FCS, with observed RNP diffusion time significantly higher than that for dual-guide RNA alone (2.0 ms versus 1.0 ms, *P*-value = .0004, Fig. 1B). The nuclear concentration of the RNP condition was higher than that observed for the dual-guide RNA only condition (7.5 × 10^7^ Cas9 per cell, 25 nM versus 19 nM, Supplementary Fig. S1C), possibly due to both nuclear localization and RNA stabilization that results from Cas9 binding [52]. Two-component analyses of nuclear autocorrelation functions obtained by FCS provided best fits to the data and were used for all future nuclear delivery analyses (Supplementary Fig. S1D and E).

Next, we quantified the nuclear concentration of Cas9 RNA containing a *B2M*-targeting, fluorescently labeled dual-guide RNA, across a range of dosages (15 × 10^7^ to 0.24 × 10^7^ Cas9 per cell) in HeLa cells at 24 h (Fig. 1C and D). The RNP nuclear concentrations resulting from electroporation were linear across this dose range (*R*^2^ = 0.96) ranging from 39 to 3 nM (Fig. 1C). Using published estimates of HeLa nuclear volume, ∼690 μm^3^ [46], we calculated 16 000–1200 Cas9 molecules per nucleus for this dose range (Fig. 1C and D, and Supplementary Table S2), which corresponds to ∼0.01% of total Cas9 per cell.

We wondered whether electroporation delivery efficiency varies substantially by cell type. Measurement of Cas9 RNP nuclear concentrations in two additional cell lines, HEK293T and U2OS, at two doses (7.5 × 10^7^ and 0.94 × 10^7^ Cas9 per cell) revealed similar values to those measured in HeLa cells (Fig. 1E). This suggests that Cas9 RNP delivery efficiency by electroporation in multiple cell types is similar and thus the large RNP dose difference required for editing in different cell types may be due to differences in epigenetic landscape or DNA repair mechanisms [53,54].

### EDVs dramatically reduce the amount of Cas9 RNP needed for genome editing

**Figure 2.**
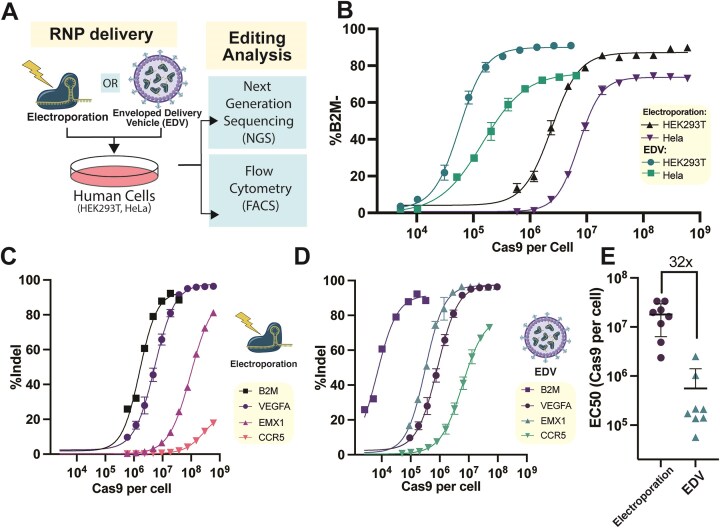
Assessing dosage requirements of Cas9 RNP delivered by electroporation and EDVs. (**A**) Experimental schematic of workflow to quantify the Cas9 RNP doses required for editing by electroporation and EDVs. (**B**) To assess Cas9 RNP dosage required for editing, HEK293T and HeLa cells were treated with varying doses of B2M-targeting Cas9 by electroporation and EDVs. Analysis was performed by flow cytometry 4 days post-treatment to assess B2M knockdown. (**C**) Electroporation and (**D**) EDV delivery of Cas9 RNP targeting the B2M, VEGFA, EMX1, and CCR5 loci in HEK293T cells. Analysis was performed by NGS 4 days post-treatment to assess indels. (**E**) Comparison of required RNP doses required for half maximal editing (EC50) delivered by electroporation and EDV for nine different B2M guides in HEK293T (*P*-value = .0003, Mann–Whitney). Analysis was performed by flow cytometry 4 days post-treatment to assess B2M knockout. *n* = 3 technical replicates were used in all experiments. Data points represent the mean with error bars displaying standard deviation (SD). RNP dose curves were modeled (Prism v10) as sigmoidal (4PL, *X* is concentration).

To determine how much functional Cas9 RNP is required for efficient editing in human cells, we first determined how much total Cas9 protein is needed for editing when delivered by electroporation or EDV into HEK293T or HeLa cells (Fig. 2A and Supplementary Fig. S2). For electroporation, the dosage that resulted in half maximal editing (EC50) of the B2M gene was 2.4 × 10^6^ and 7.3 × 10^6^ Cas9 per cell in HEK293T and HeLa cells, respectively (Fig. 2B). For EDV delivery, the EC50 was 5.7 × 10^4^ and 1.5 × 10^5^ Cas9 per cell in HEK293T and HeLa cells, respectively (Fig. 2B). These values correspond to 42- and 50-fold reductions in required Cas9 dose for HEK293T and HeLa cells, respectively, for EDV-mediated RNP delivery compared to electroporation.

Extensive work has shown that the choice of gRNA strongly impacts the activity and specificity of Cas9 [36, 55], but it remains unknown the degree to which the gRNA impacts the Cas9 RNP dosage required for editing. We compared doses required for editing using four different gRNAs targeting *B2M*, *VEGFA*, *CCR5*, and *EMX1* and found that the amount of Cas9 required for editing varied by >100-fold depending on guide choice (Fig. 2C and D).

We wondered whether these differences in required RNP dosage by these gRNAs were due to differences between the *B2M*, *VEGFA*, *CCR5*, and *EMX1* loci. Therefore, to minimize the impact of potential differences in chromatin state, we generated a panel of nine gRNAs targeting a ∼200-bp window in the *B2M* locus and compared the dosages required for *B2M* knockout in HEK293T and HeLa cells (Fig. 2E and Supplementary Fig. S3). Different gRNAs resulted in substantial differences (>100-fold) between RNP doses required for editing using either electroporation or EDVs. For electroporation and EDVs, respectively, the EC50 values across gRNAs were highly correlated between cell lines (*R*^2^ = 0.78 and *R*^2^ = 0.83) (Supplementary Fig. S4A and B). Interestingly, however, the gRNA trends were only loosely correlated across delivery modalities (*R*^2^ = 0.55, Supplementary Fig. S4C) and there was no correlation between individual gRNAs’ maximum editing levels and dosage requirements (*R*^2^ = 0.01 and *R*^2^ = 0.08, Supplementary Fig. S4D and E).

To remain functional in human cells, Cas9 must remain guide-complexed and retain biochemical cleavage activity. Typically, ∼20%–40% of purified Cas9 is active *in vitro* [56–58]. Across three gRNAs, our in-house purified Cas9 averaged 30% activity (Supplementary Fig. S5), but we wondered whether EDV packaging impacts the activity of encapsulated Cas9 RNPs. By Cas9 and p24 ELISA, we determined that each EDV packages 273 ± 52.5 Cas9 molecules (Supplementary Figs S2 and S6A; see the “Materials and methods” section). Next, we quantified the maximum percent of Cas9 protein in EDVs that could be complexed with sgRNA by measuring the Cas9 protein and sgRNA concentrations using Cas9 ELISA and RT-qPCR, respectively (Supplementary Fig. S6B). Across five independent batches of EDVs, only 8.3 ± 1.7 sgRNA molecules were measured for every 100 molecules of Cas9 protein (Supplementary Fig. S6B).

We first tested whether sgRNA availability limits the Cas9 cleavage functionality in EDVs. Cas9 packaged in EDVs with or without sgRNA was introduced into cells that were also transfected with a plasmid expressing a *B2M*-targeting sgRNA (Supplementary Fig. S6C). However, this additional gRNA supplementation failed to result in measurable improvement in editing efficacy (Supplementary Fig. S6C). We next tested whether the Cas9 protein in EDVs is degraded or unfolded, which could render it incapable of gRNA binding. Using western blotting, we determined that most Cas9 in EDVs remains uncleaved from the lentiviral polyprotein Gag or is degraded in EDVs, and that only 35% of the Cas9 is intact (Supplementary Fig. S6D). Together with previous results showing that Cas9 protein alone readily denatures at 37°C [59], this finding suggests that the vast majority of Cas9 in EDVs is not functional. Furthermore, we conclude that the per-molecule difference in RNP delivery efficiency between EDVs and electroporation is substantially greater than the >30-fold difference measured in editing assays (Fig. 2).

### EDV-mediated Cas9 RNP delivery results in rapid DNA cleavage and repair

**Figure 3.**
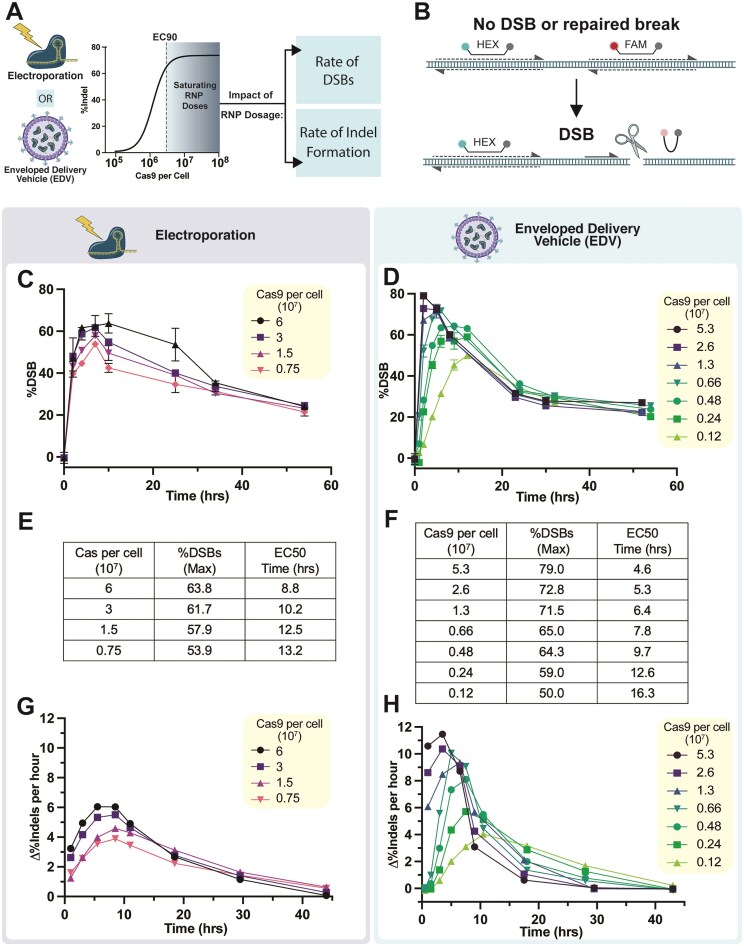
Kinetics of double-strand breaks and DNA repair resulting from delivery by RNP electroporation and EDVs. (**A**) Schematic overview of time course experiment comparing the impact of saturating doses, defined as doses at or exceeding levels for 90% of the maximum editing (>EC90), of Cas9 RNP delivered by electroporation and EDVs on the rate of double-strand breaks and consequent indel repair detected by NGS. (**B**) Experimental setup of the ddPCR used to quantitatively detect DSBs. An HEX probe spans the first amplicon that is centromere proximal to the break site. A second FAM probe anneals to the second amplicon, which is lost upon DSB or chromosome loss. DSBs caused by (**C**) electroporation and (**D**) EDV delivery of Cas9 RNP targeting the B2M locus over a 54-h time frame. *t* = 0 time points represent the baseline readout prior to Cas9 delivery by electroporation or EDVs. Tables comparing the maximum percentage of synchronous DSBs and time frame for half maximal editing (EC50) resulting from delivery of *B2M*-targeting RNP in HEK293T by (**E**) electroporation and (**F**) EDV delivery. Rate of indel formation caused by (**G**) electroporation and (**H**) EDV delivery of *B2M*-targeting RNP in HEK293T cells measured by NGS (raw NGS time courses in Supplementary Fig. S7). All doses are at levels that meet or exceed the amount necessary for 90% maximal editing. *n* = 3 technical replicates were used in all experiments. Data points represent the mean with error bars displaying SD.

The efficiency of genome editing depends on the integrated amount and duration of functional Cas9 RNP residing in the cell nucleus. To gain insight into the rate and duration of Cas9 RNP activity as a function of delivery modality, we treated cells with a range of saturating dosages (defined as that required for 90% maximal editing) of *B2M*-targeting RNP delivered by either electroporation or EDVs (Fig. 3A). Cells were harvested over the course of 54 h post-treatment and analyzed using ddPCR and NGS to measure DSBs and genome edits, respectively (Fig. 3A and B). Electroporation permeabilizes mammalian cells using electric pulses lasting a few microseconds to induce pore formation in the plasma membrane. These pores reseal within seconds to minutes, however, limiting the time in which RNPs can access the cell interior [60]. Consistent with this transient delivery window, at all RNP amounts tested, electroporation resulted in the majority of observed DSBs occurring within 2 h post-delivery (Fig. 3C). The highest and lowest (6 × 10^7^ and 0.75 × 10^7^ Cas9 per cell) RNP doses resulted in similar maximum concurrent DSBs (64% versus 54%, respectively) (Fig. 3C).

We initially hypothesized that EDV delivery would take longer than electroporation because EDV endocytosis requires fusion with endosomes to deliver RNPs into the cytosol. Interestingly, at a high RNP dose (5.3 × 10^7^ Cas9 per cell), EDV-mediated RNP delivery occurred as quickly as electroporation, resulting in most DSBs occurring within 2 h (Fig. 3D). These data show that the minimal time frame for EDV-mediated Cas9 RNP intracellular delivery, nuclear localization, and genome target cleavage occurs within 2 h, despite requiring additional delivery steps. Notably, for EDVs the time frame for DSB formation was substantially more dose dependent, with the lowest doses of Cas9 delivered by EDV requiring 12 h to reach the maximum level of DSBs (49%) (Fig. 3D). Importantly, this shows that it is possible to control and tune the rate of editing with the concentration of EDV.

In the absence of a DNA donor template, DSBs are typically resolved through nonhomologous end joining. It remains unclear whether the time frame for DNA repair is impacted by delivery mode, particularly because delivery can perturb cellular metabolism and viability [19, 60]. At high RNP doses (6 × 10^7^ and 5.3 × 10^7^ Cas9 per cell), both strategies result in similar time frames for DSB formation, but EDV delivery generated genome edits twice as fast (Fig. 3E–H and Supplementary Fig. S7). At these same doses, half maximal editing for electroporation and EDVs occurred by 8.8 and 4.6 h, respectively (Fig. 3E and F, and Supplementary Fig. S7). For electroporation, genome editing occurred within a similar time frame for all doses, with the maximum rate of editing occurring in the 6–8-h window (Fig. 3G). Conversely, the rate of editing for EDVs was highly dose dependent (Fig. 3H). At the earliest time point (1 h), the rate of genome edits was three times higher for EDVs than for electroporation at comparable doses, 10.6% edits/h versus 3.2% edits/h, respectively (Fig. 3G and H, and Supplementary Fig. S7). Combined, these results suggest that EDVs can rapidly deliver Cas9 into the cell interior and may continue to deliver functional RNPs into the nucleus over a prolonged time period.

One advantage of RNP delivery is increased editing specificity resulting from its transient activity within the cell. Therefore, we investigated whether the extended RNP delivery time by EDVs impacted editing specificity compared to electroporation. Using targeted deep sequencing, we compared editing specificity for two guides, targeting *VEGFA* and *EMX1*, at their on-target site and previously validated guide-seq off-target sites [61]. We titrated RNP doses delivered by electroporation and EDV, and compared the editing specificity by calculating the ratio of measured on-target editing compared to off-target editing. Consistent with previous research [3–5], RNP delivery by either strategy resulted in improved editing specificity compared to extended lentiviral expression (Supplementary Fig. S8). Nuclease activity at mismatched off-target sequences is kinetically slower than that at fully matched complementary on-target sequences [62]. Analogously, for both electroporation and EDV delivery, on-target editing occurred at substantially lower RNP dosages than off-target edits (Supplementary Fig. S8A–D). Importantly, editing specificity was highly dependent on RNP dosage, with specificity dramatically decreasing in slight excess of doses required for half maximal on-target editing (Supplementary Fig. S8A–D). For example, at RNP doses sufficient for 50% on-target editing at the EMX1 loci, the on:off-target ratio was 7.7 and 6.9 for electroporation and EDVs, respectively. At four-fold excess of these dosages, resulting in 80.9% and 83.8% editing, the on:off-target ratio was 15.3 and 3.6 for electroporation and EDVs, respectively (Supplementary Fig. S8E and F).

Interestingly, EDV delivery did lead to decreased specificity at both target sites compared to electroporation. For VEGFA, the maximum on:off-target ratio was 8.5 and 7.3 for electroporation and EDVs, respectively (Supplementary Fig. S8E and F). For EMX1, the specificity difference between delivery modalities was more pronounced, with a maximum on:off-target ratio of 15.8 and 8.0 for electroporation and EDVs, respectively (Supplementary Fig. S8E and F). We hypothesize that the difference in specificity between these two RNP delivery strategies may result from increased EDV persistence in the cell.

### EDV delivery results in extended nuclear accumulation of Cas9 RNPs over time

**Figure 4.**
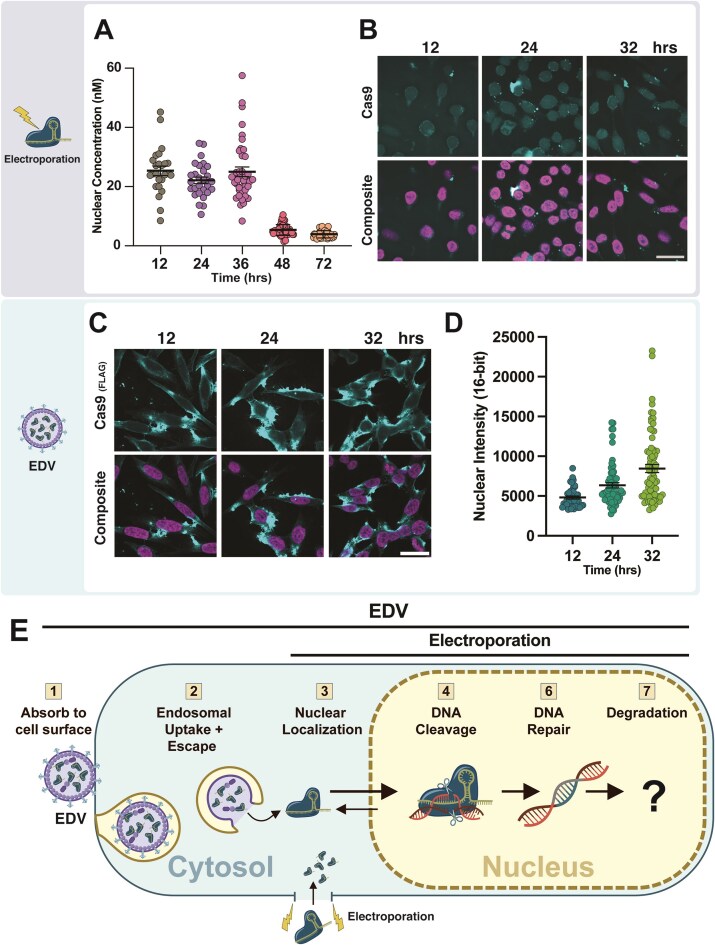
Comparison of nuclear localization and concentration of Cas9 delivered by electroporation and EDVs as function of time. (**A**) Quantification of the nuclear concentration of Cas9 RNP in HeLa cells by FCS at 12, 24, 36, 48, and 72 h post-electroporation (7.5 × 10^7^ Cas9 per cell). Each point represents the concentration in an individual cell. FCS values are provided in nM; *n* > 25 for each FCS condition with at least two biological replicates each (mean ± SEM). All concentration values and diffusion times were derived by fitting FCS traces with a two-component 3D diffusion equation (see the “Materials and methods” section). Representative fixed confocal images of HeLa cells stained for Cas9 (top) and composite image of Cas9 overlaid with nuclear counterstain (SYTOX™ Deep Red Nucleic Acid Stain) showing the nuclear intensity of Cas9 at 12, 24, and 36 h following (**B**) electroporation (1.2 × 10^8^ Cas9 per cell) and (**C**) EDV treatment (∼3.0 × 10^6^ Cas9 per cell). Scale bar is 25 μm. (**D**) Relative quantification of median nuclear intensity of Cas9 delivered by EDV at 12, 24, and 36 h (see the “Materials and methods” section). Intensity on 16-bit scale (0–65 536). Each data point represents an individual nuclei, *n* > 40 (mean ± SEM). (**E**) Schematic overview of Cas9 delivery by EDVs and electroporation.

We hypothesized that the difference in genome editing kinetics observed using electroporation versus EDV-mediated Cas9 RNP delivery may correspond to the length of time that Cas9 RNPs reside in the nucleus. We hypothesized that EDVs could continue to deliver Cas9 RNPs to the nucleus over an extended time frame, whereas electroporation would result in decreasing levels of nuclear Cas9 following initial delivery. To quantify electroporated Cas9 RNPs as a function of time, we used FCS to measure the nuclear concentration of Cas9 RNP in HeLa cells at 12, 24, 36, 48, and 72 h following electroporation (Fig. 4A). Following electroporation (7.5 × 10^7^ Cas9 per cell), nuclear RNP concentrations did not vary from 12 to 36 h but dropped by >75% by 48 h (Fig. 4A and Supplementary Table S2). We used confocal microscopy to visualize the electroporated Cas9 RNPs in HeLa cells at these time points and validated the results by quantifying relative nuclear concentrations of Cas9 using fixed confocal microscopy (Fig. 4B and Supplementary Fig. S9A; see the “Materials and methods” section). The electroporated Cas9 exhibited punctate staining, which can be indicative of aggregation, endosomal entrapment, or other forms of intracellular sequestering [31,44,45]. To determine whether a significant proportion of electroporated RNP remained trapped in endosomes, we stained for Rab5a and LAMP1, an early stage endosomal and lysosomal marker, and found minimal evidence for Cas9 colocalization inside endosomes or the lysosome (Supplementary Fig. S9B–E). In addition, gRNA spacer sequence had no measurable impact on nuclear concentrations following electroporation with Cas9 complexed with a high- or poor-performing gRNA (Supplementary Fig. S9F). Consistent with our editing time course (Fig. 3), the nuclear concentration of Cas9 delivered by EDVs continued to increase for up to 32 h after delivery (Fig. 4C and D, and Supplementary Fig. S10A). Interestingly, for EDV delivery, Cas9 appeared to also accumulate around the nuclear envelope, which was not observed for electroporation (Fig. 4B, Supplementary Fig. S10B and C, and Supplementary Table S3). This may be due to the nuclear export signals (NESs) present on the uncleaved Gag-Cas9 construct that facilitates EDV packaging (Supplementary Figs S6 and S10B and C) [26, 63] causing some of the Cas9 to accumulate around the nuclear envelope.

Collectively, these results together with insights from previous studies support a model for how EDV delivery impacts the rate and mode of Cas9 RNP delivery (Fig. 4E). The earliest detectable genome editing occurred within 2 h for all EDV doses studied (Fig. 3), demonstrating that Cas9 RNP nuclear entry occurs quickly following delivery. EDVs adsorb quickly to the cell membrane [64] and remain associated even after cell washing. Over time, these EDVs undergo endosomal uptake and escape mediated by VSVG on the EDV surface [64]. Cas9 RNP encapsulation by EDVs appears to extend RNP half-life in the nucleus, possibly by impacting Cas9 degradation mechanisms.

## Discussion

Safe and effective delivery of CRISPR–Cas9-based genome editing enzymes has profound potential to advance both therapeutic development and fundamental research. Focusing on Cas9 RNP delivery, we compared electroporation and EDVs for their ability to introduce sufficient RNPs into cells to mediate intended genome modifications. Our data show that a minimum of ∼1300 Cas9 molecules per cell nucleus are required for half maximal genome editing across a range of different human cell lines. We also found that increasing genome editor lifetime in the nucleus is critical to minimize effective RNP concentration while maintaining editing efficacy. Recent work demonstrated that inhibiting Cas9 Keap1-mediated degradation enhanced epigenome editor performance [65]. Future research should investigate whether thermostable Cas9 variants, such as iGeoCas9 [59], exhibit increased nuclear half-life or, for EDV delivery, allow for a larger proportion of RNPs to be functional.

We found that packaged delivery of Cas9 RNPs within EDVs resulted in continued RNP nuclear localization over a prolonged period (Fig. 4C and D). Similarly, other RNP delivery strategies, including related virus-like particles [21, 24, 25, 66, 67], lipid nanoparticles [59, 68, 69], and cell-penetrating peptides [19, 70], may result in prolonged delivery windows compared to direct RNP electroporation. Importantly, these extended time windows could be leveraged for spatiotemporal control [28] or to impact DNA repair outcomes [19, 28]. Furthermore, the delay between DSB and subsequent genome editing will likely be cell-type dependent, with longer repair times expected for clinically relevant post-mitotic cell types such as neurons and cardiomyocytes [28].

Improving nuclear localization efficiency of the Cas9 RNP is of paramount importance. Our study indicates that EDV delivery protects the Cas9 RNP and increases its delivery duration. For EDV delivery, there was noticeable accumulation of Cas9 around the nuclear envelope, which suggests that nuclear localization may be limiting. This may be partially due to the NESs added to the gag polyprotein to facilitate RNP packaging [21, 26, 66] and is consistent with recent EDV engineering demonstrating that adding additional nuclear localization signals (NLSs) to Cas9 can improve EDV-mediated editing efficiency by ∼2-fold [63]. Interestingly, there appears to be an inherent trade-off between requiring cytosolic localization for efficient EDV production, with additional NLSs reducing RNP packaging, and effective nuclear localization necessary for editing following EDV delivery [63]. For RNP electroporation, previous studies have also illustrated the importance of NLS optimization [19, 63]. The optimal NLS configuration is different for other type II and type V Cas nucleases [71,72], such as Cas12a that contains putative NESs [73], and may also differ for fusion constructs (i.e. base or prime editors). In addition, the impact of NLS tiling on cellular perturbation, editing specificity, and half-life remains to be tested.

We show that understanding the impact of delivery modality on RNP intracellular trafficking, localization, and genome editing efficacy can identify delivery bottlenecks that could be the focus of future engineering of improved RNP-based gene therapies. Our study examined the dosage requirements of nuclease active SpyCas9 in human cell lines. Future work should investigate the editor dosage requirements for other clinically relevant cell types, as well as whether or how these requirements differ for other editing tools used for base, prime, and epigenome editing, to best preserve genomic and cellular integrity while efficiently achieving the desired genomic alterations.

## Supplementary Material

gkaf105_Supplemental_Files

## Data Availability

Flow cytometry, FCS, unprocessed ddPCR data, quantified confocal *Z*-stacks, and NGS raw files are available upon request. All other data are provided in the main text or supplementary materials.
