# Supplementary material for: Packaged delivery of CRISPR–Cas9 ribonucleoproteins accelerates genome editing
Source: Nucleic Acids Res. 2025 Feb 27;53(5):gkaf105. doi: 10.1093/nar/gkaf105 (PMC11878570; doi:10.1093/nar/gkaf105)
Supplement: gkaf105_Supplemental_Files [file gkaf105_supplemental_files.zip › 241212_Revision_Manuscript_Supp.pdf]

Supplementary Material

Supplemental Figures 1-10
Supplemental Table 1-3

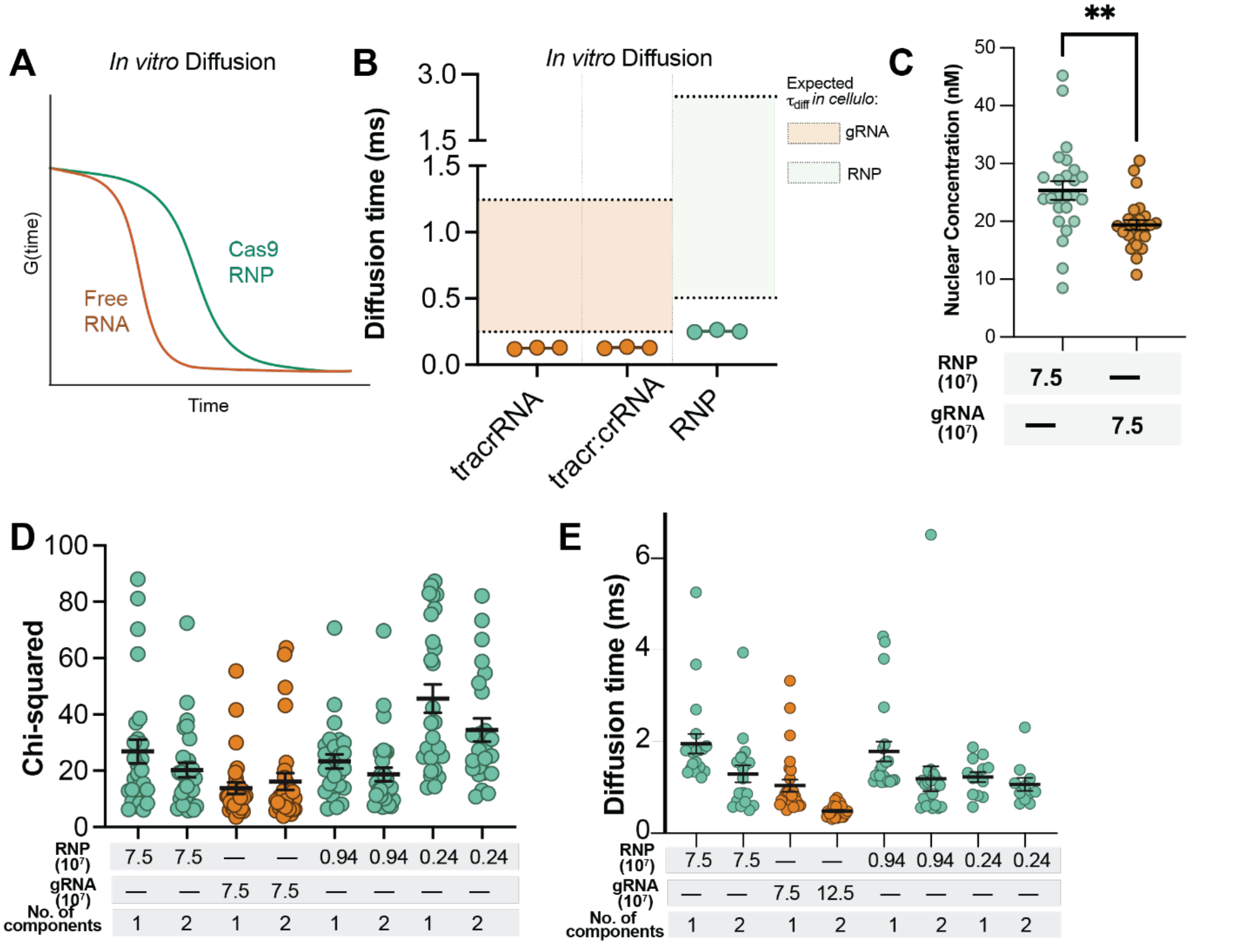

Supplemental Figure 1: Optimization of fitting parameters for measuring Cas9 RNP nuclear concentration by fluorescence correlation spectroscopy.
FCS measurements produce an autocorrelation function, which can be fit to derive both the concentration of delivered material and its diffusion time(s). **(A)** Example FCS traces for free sgRNA versus an intact Cas9 RNP. The diffusion time is calculated as the inflection point of the autocorrelation function. **(B)** FCS traces were measured for tracrRNA, tracr:crRNA, and Cas9 RNP *in vitro* (right). Samples were prepared at a final concentration of 400 nM in DMEM. Each point represents the average diffusion time (t_{diff}) calculated from ten autocorrelation traces at that point. Average t_{diff} for tracrRNA = 0.126 ms, for sgRNA = 0.129 ms, and for Cas9 RNP = 0.253 ms. **(C)** HeLa cells were electroporated with fluorophore labeled gRNA (ATTO™ 550-tracrRNA:crB2M (IDT)) or with RNP pre-complexed 1:1 with fluorophore labeled guide (7.5 x10^7 Cas9 RNP or gRNA per cell). FCS diffusion times provided in ms, n>25 for each FCS condition with at least two biological replicates each (mean ± SEM). **(D)** and **(E)** Comparison of one-component and two-component diffusion fitting equations for FCS data in cells. **(D)** Chi-square (c^2) values for autocorrelation curves fitted with one- or two-component diffusion

equations plotted as a function of RNP or sgRNA dosage. Each point represents the c^2 for the fitting of an average autocorrelation curve (calculated as the mean autocorrelation function from ten individual traces) obtained from an individual cell. **(E)** Diffusion times for the autocorrelation curves fitted in **(D)**. For a one-component diffusion equation, only one diffusion time is produced. For a two-component diffusion equation, a fast diffusion and a slow diffusion time are both calculated; the fast diffusion time is plotted here. Each point corresponds to the t_{diff} derived from each fitted autocorrelation curve in **(D)**.

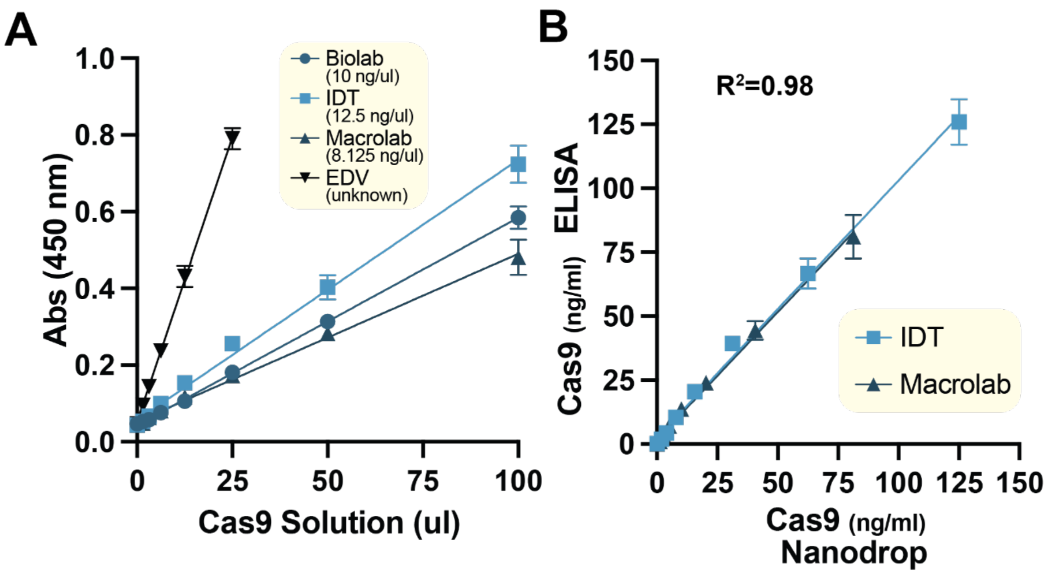

Supplemental Figure 2: Validation of ELISA for measuring Cas9 used for electroporation and in EDVs

(A) Absorbance values from ELISA for Cas9 as purified RNP diluted 800-fold, from commercial (IDT) and in-house source (UC-Berkeley, Macrolab, QB3), and 20x concentrated EDVs prepared by ultracentrifugation diluted 4-fold (n=3). **(B)** Absorbance values from ELISA used to quantify concentration of Cas9 in commercial (IDT) and in-house (UC-Berkeley, Macrolab, QB3) samples compared to reported concentrations calculated from photospectrometry (Nanodrop) (R^2=0.98, n=3).

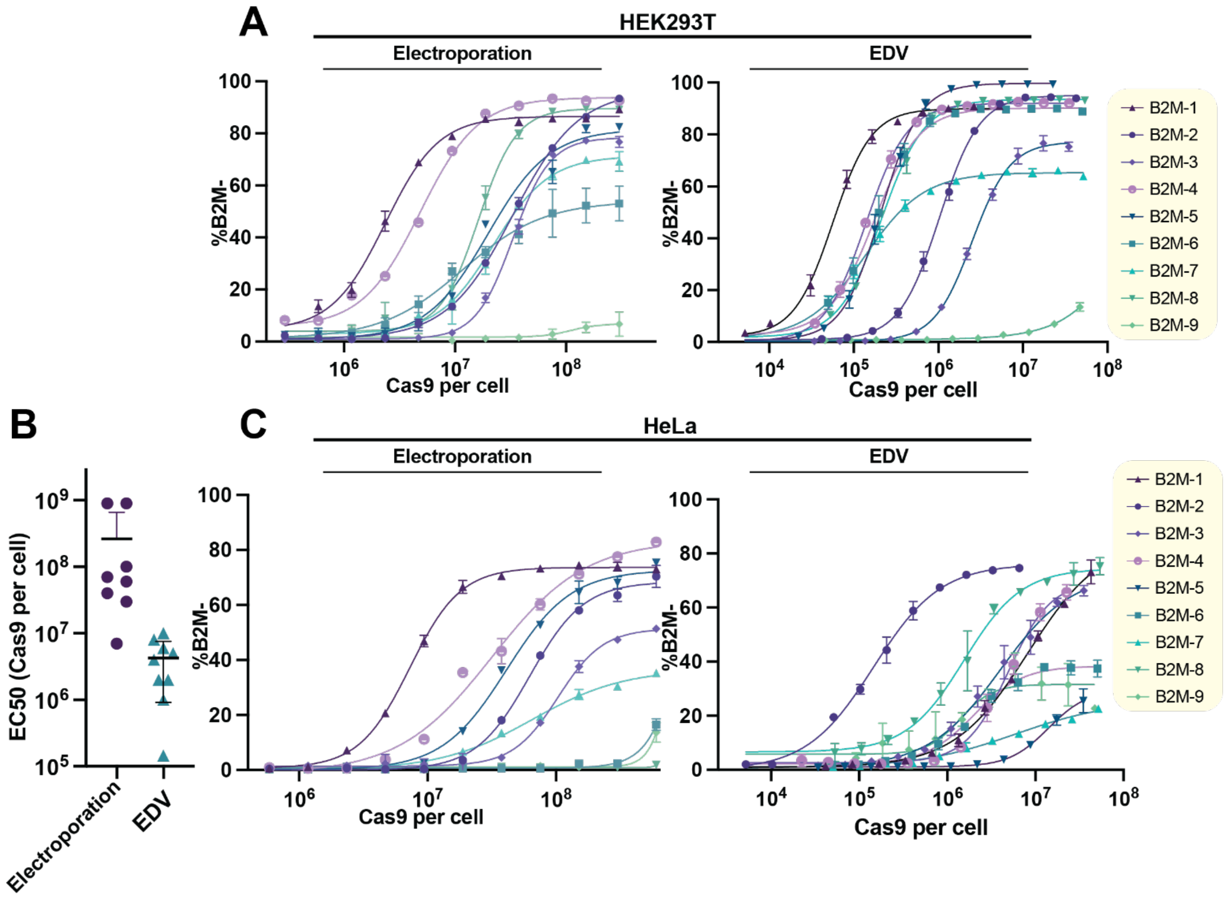

Supplemental Figure 3: Cas9 RNP doses required for editing by electroporation and EDVs

(A) B2M editing by Cas9 RNP delivered by (Left) electroporation and (Right) EDV with 9 different B2M-targeting guides in HEK293T. **(B)** Comparison of Cas9 RNP doses required for half maximal editing (EC50) when delivered by electroporation and EDVs in HeLa cells. **(C)** HeLa cells treated with Cas9 RNP complexed with the same 9 guides as in **(A)** delivered by (Left) electroporation and (Right) EDVs. Cells were analyzed by flow cytometry on day 4 (n=3). Datapoints represent the mean with error bars displaying SD. RNP dose curves were modeled (Prism v10) as sigmoidal (4PL, X is concentration).

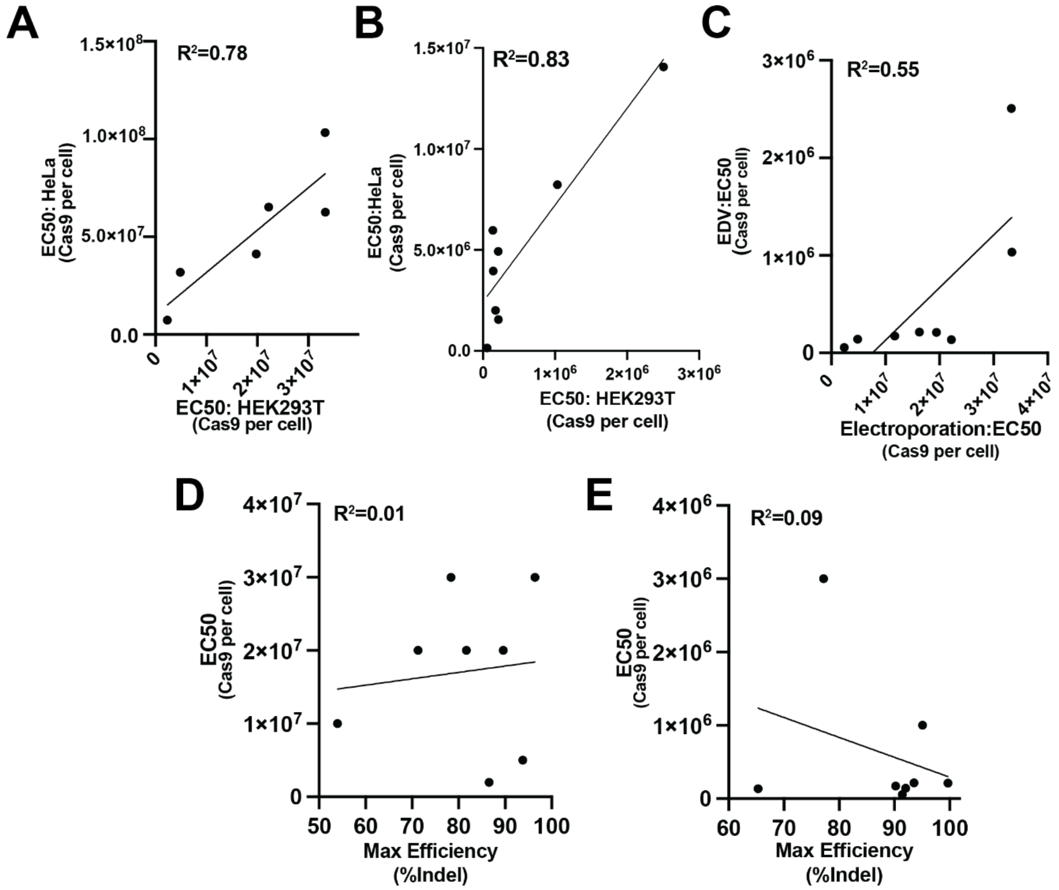

Supplemental Figure 4: Analyzing the linear relationships between RNP delivery modalities and guide RNA on editing dosage requirements

Correlation analysis of dosage required for half maximal editing (EC50) from Cas9 RNP delivered by **(A)** Electroporation and **(B)** EDVs in HEK293T (x-axis) and HeLa cells (y-axis).**(C)** Correlation analysis of dosage required for half maximal editing (EC50) from Cas9 RNP delivered by electroporation (x-axis) versus EDV (y-axis). Relationship between gRNA maximum editing efficiency (i.e. the top plateau of dosage curve) versus the dosage EC50 for **(D)** electroporation and **(E)** EDV in HEK293T cells. For correlation analyses, each value calculated from HEK293T HeLa dose curves (Supplemental Figure 3). n=3 technical replicates were used in all experiments.

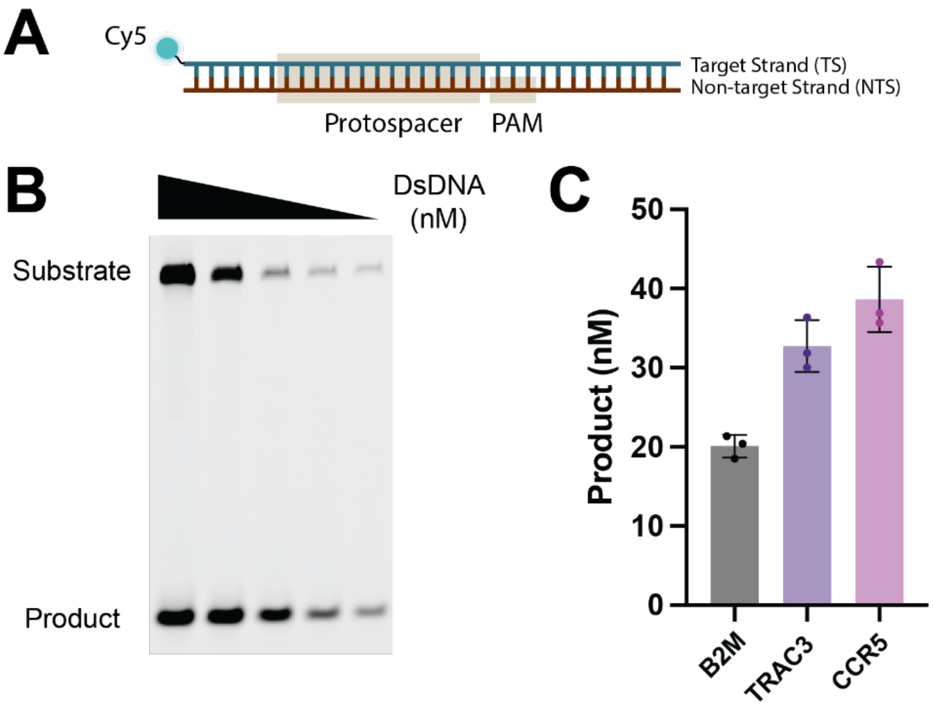

Supplemental Figure 5: In vitro cleavage assay to assess percent activity of purified Cas9
(A) Schematic of double-stranded DNA (dsDNA) substrate used for assessing the biochemical cleavage activity of purified Cas9 RNP, a single turnover enzyme (1), by active site titration. The Cy5 fluorophore label is on the 5' end of the target strand. **(B)** Representative gel showing 100 nM Cas9 RNP, complexed with TRAC3 guide, incubated with annealed DsDNA substrate with TRAC3 active site at titrated concentrations. Percent cleavage calculated by same method as (2) **(C)** Comparison of cleavage activity of in-house (UC-Berkeley, Macrolab, QB3) Cas9, complexed with respective sgRNA (IDT), targeting substrates with spacers from B2M, TRAC3, CCR5. n=3 technical replicates were used in all experiments. Datapoints represent the mean with error bars displaying SD. Oligos used are in Supplemental table 1.

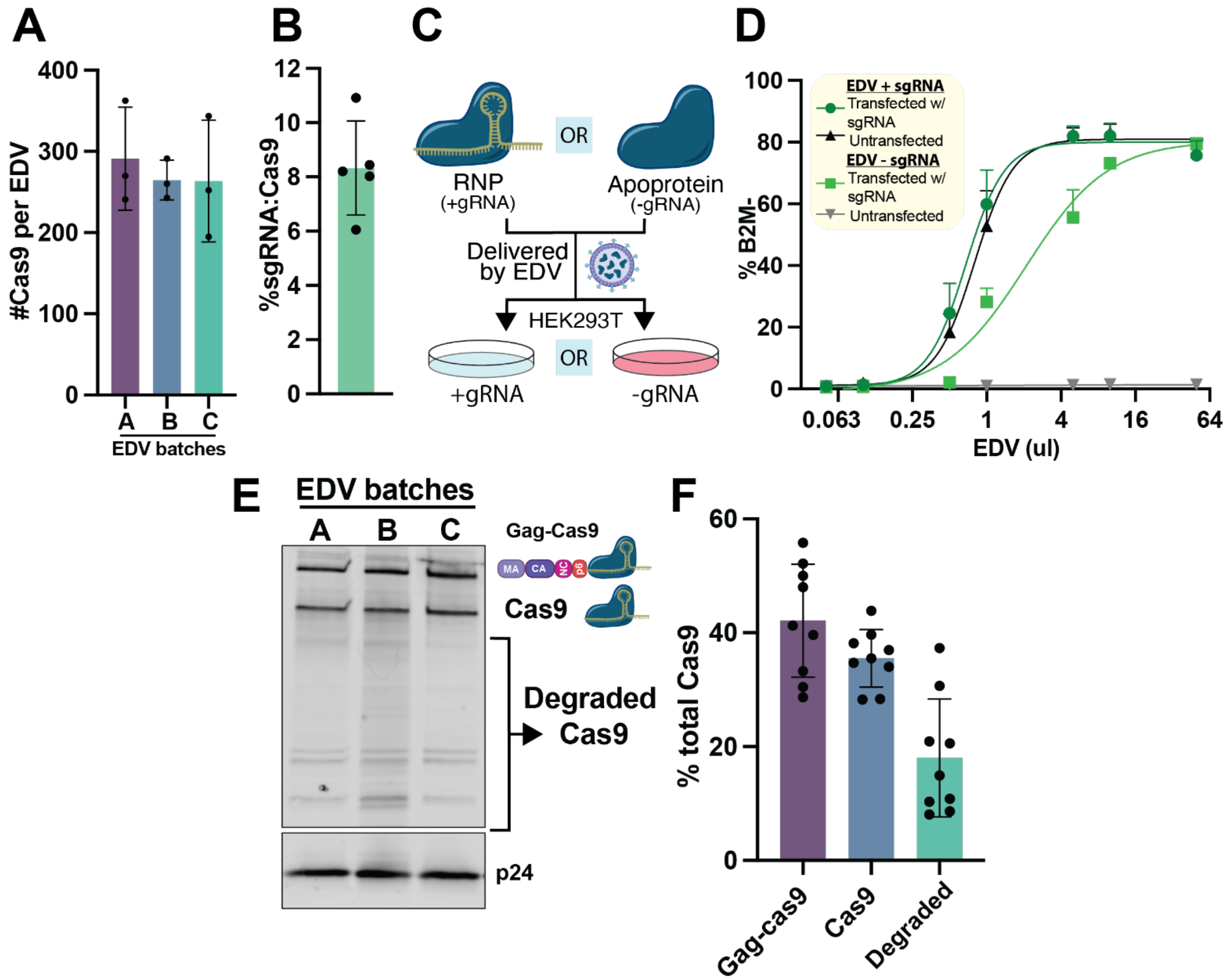

Supplemental Figure 6: Determining the percent of RNPs in EDVs that are functional
(A) Quantification of the number of Cas9 molecules per EDV. p24, the capsid protein and a key structural component of EDVs and native HIV, and Cas9 were quantified by ELISA (n=3 biological replicates measured in triplicate). There are 273 +/- 52.5 Cas9 per EDV. **(B)** The percent of B2M sgRNA available relative to Cas9 protein molecules in EDVs (n=5 biological replicates, mean depicted from 3 technical replicates). The molecules of intact B2M sgRNA and Cas9 protein were determined by quantitative RT-PCR and Cas9 ELISA (n=5 biological replicates measured in triplicate), respectively. **(C)** Schematic of experimental setup to determine whether sgRNA is limiting editing potency of EDVs in cells. HEK293T cells, either untreated or transfected with U6-B2M-sgRNA expression plasmid, were treated with EDVs containing *B2M*-targeting Cas9 RNP or Cas9 apoprotein (n=3). **(D)** B2M knockout was assessed by flow cytometry four days post treatment with minimal difference between conditions where HEK293T cells expressed excess B2M-targeting gRNA versus delivery with EDVs containing *B2M*-targeting Cas9 RNP. **(E)** Western blot of 20x ultracentrifuged concentrated EDVs for Cas9 and p24 (loading control). **(F)** Densitometry analysis of the percent of total cas9 in EDVs that remains uncleaved from gag-Cas9, free Cas9, and major Cas9 degradation products. Datapoints represent the mean with error bars displaying SD.

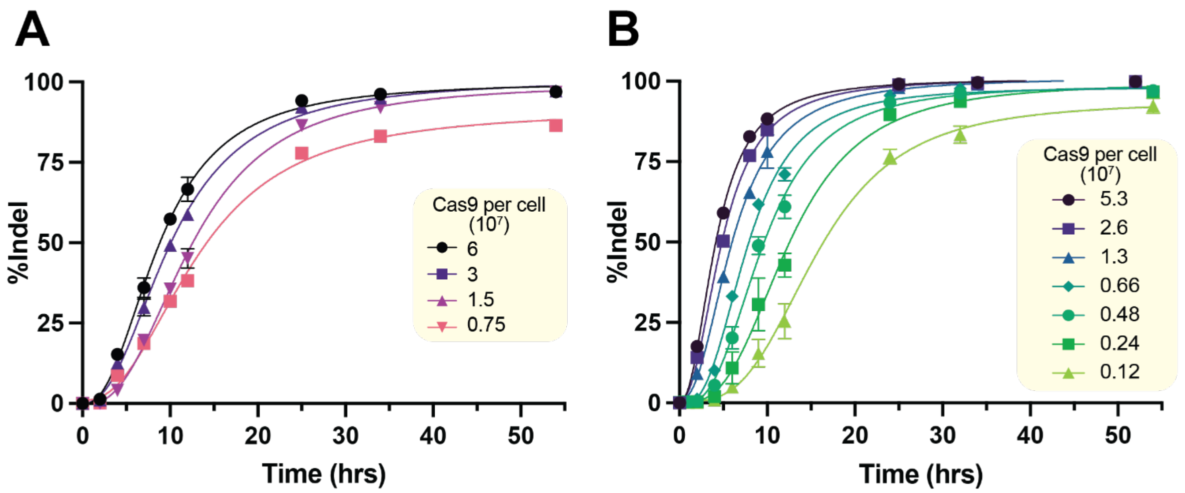

Supplemental Figure 7: Indels resulting from Cas9 delivery by electroporation and EDVs
Indel formation caused by **(A)** electroporation and **(B)** EDV delivery of *B2M*-targeting RNP in HEK293T cells measured by NGS. All doses are at levels that meet or exceed the amount necessary for 90% maximal editing. The first derivative (rate of indel formation) of these curves is shown in Fig. 3G-H for electroporation and EDV delivery. n=3 technical replicates were used in all experiments. Datapoints represent the mean with error bars displaying SD.

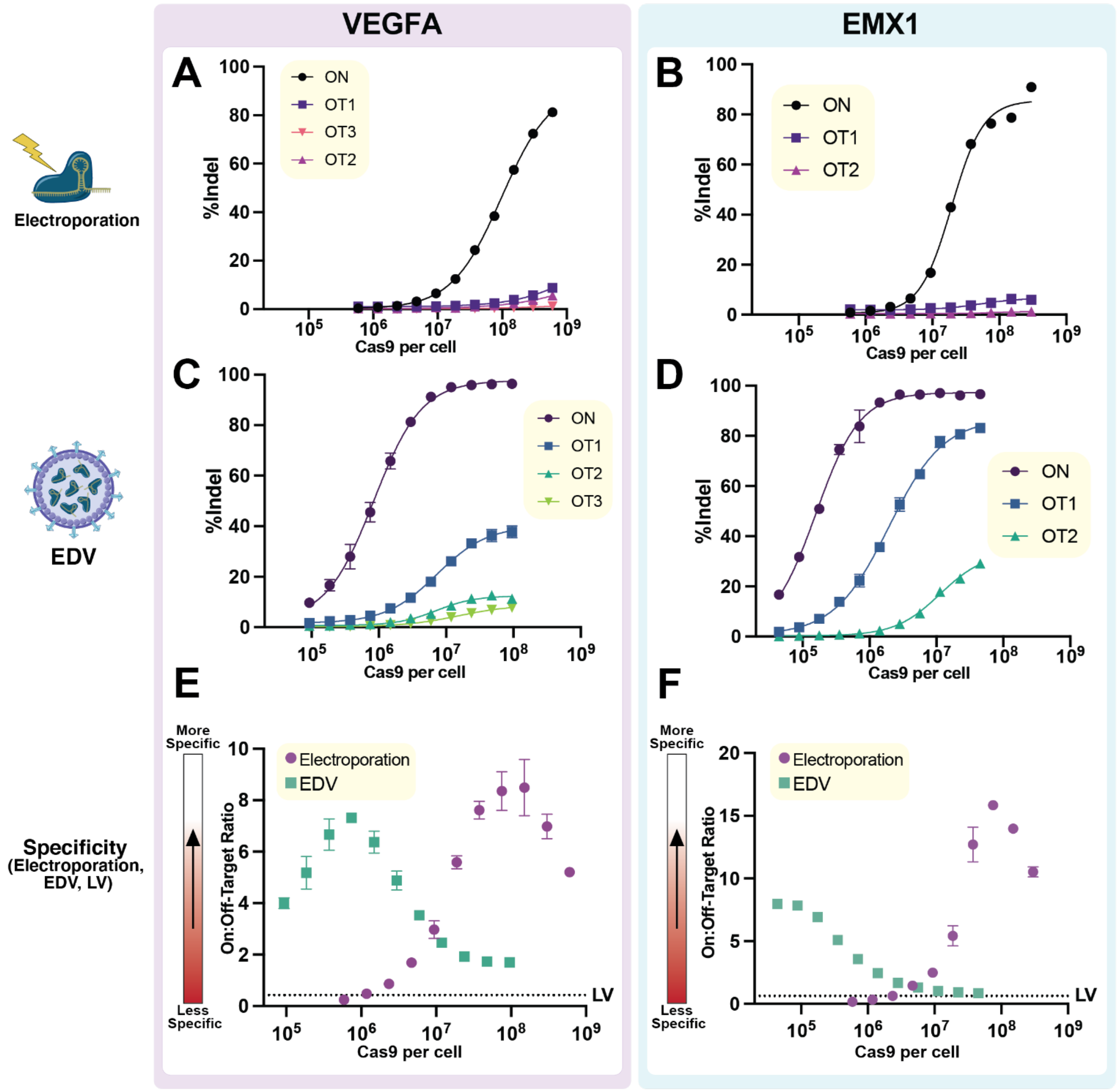

Supplementary Figure 8: Editing specificity resulting from delivery by RNP electroporation and EDVs

On-target and previously established (3) off targets were analyzed by targeted deep sequencing to compare relative editing specificity. Indels resulting from titrated RNP dosages delivered by electroporation targeting **(A)** VEGFA and **(B)** EMX1 at on-target and known off-target sites. Indels resulting from titrated RNP dosages delivered by EDVs targeting **(C)** VEGFA and **(D)** EMX1 at on-target and off-target sites (ON = on-target, OT = Off-target). Comparison of the ratio of on-target indel frequency to the combined off-target indels for lentivirus (LV) versus titrated RNP doses delivered by electroporation and EDVs targeting **(E)** VEGFA and **(F)** EMX1. n=3 technical replicates were used in all experiments. Datapoints represent the mean with error bars displaying SD. NGS primers used for target deep sequencing listed in Supplemental Table 1.

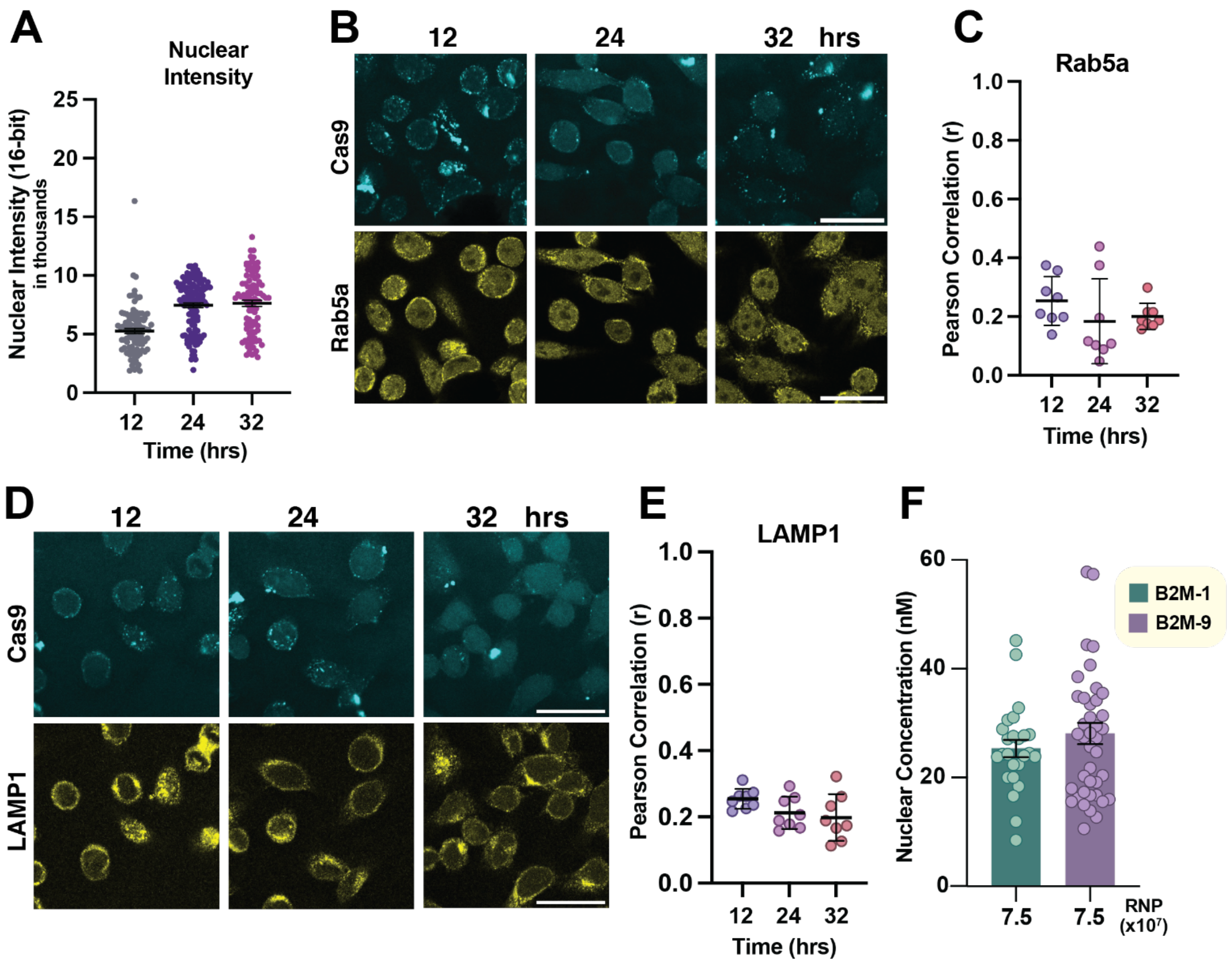

Supplemental Figure 9: Confocal microscopy for relative quantification of electroporated Cas9 RNP in nuclei and endosomes.

(A) Relative quantification of median nuclear intensity of Cas9 delivered by electroporation at 12, 24 and 32 hours in HeLa cells. (Representative images in Fig. 4B). Intensity on 16-bit scale (0 to 65536). Each data point represents an individual nuclei n> 94 (mean ± SEM). (see Methods for details). **(B)** Electroporated HeLa cells at 12, 24 and 32 hours stained to image Cas9 (Above) and Rab5a, an early endosome marker (Below). **(C)** Quantification of the colocalization of Cas9 and Rab5a at 12, 24 and 32 hours. **(D)** Electroporated HeLa cells at 12, 24 and 32 hours stained to image Cas9 (Above) and LAMP1, a lysosomal marker (Below). **(E)** Quantification of the colocalization of Cas9 and LAMP1 at 12, 24 and 32 hours. Each data point represents an independent z-stack (n=8 per time point) quantified with Pearson's correlation analysis (HuygensPro, see Methods for details). **(F)** Comparison of nuclear concentration of electroporated Cas9 ($7.5x10^7$) complexed with high- and poor- performing gRNA (Supplemental Table 1) 24 hours following electroporation by FCS.

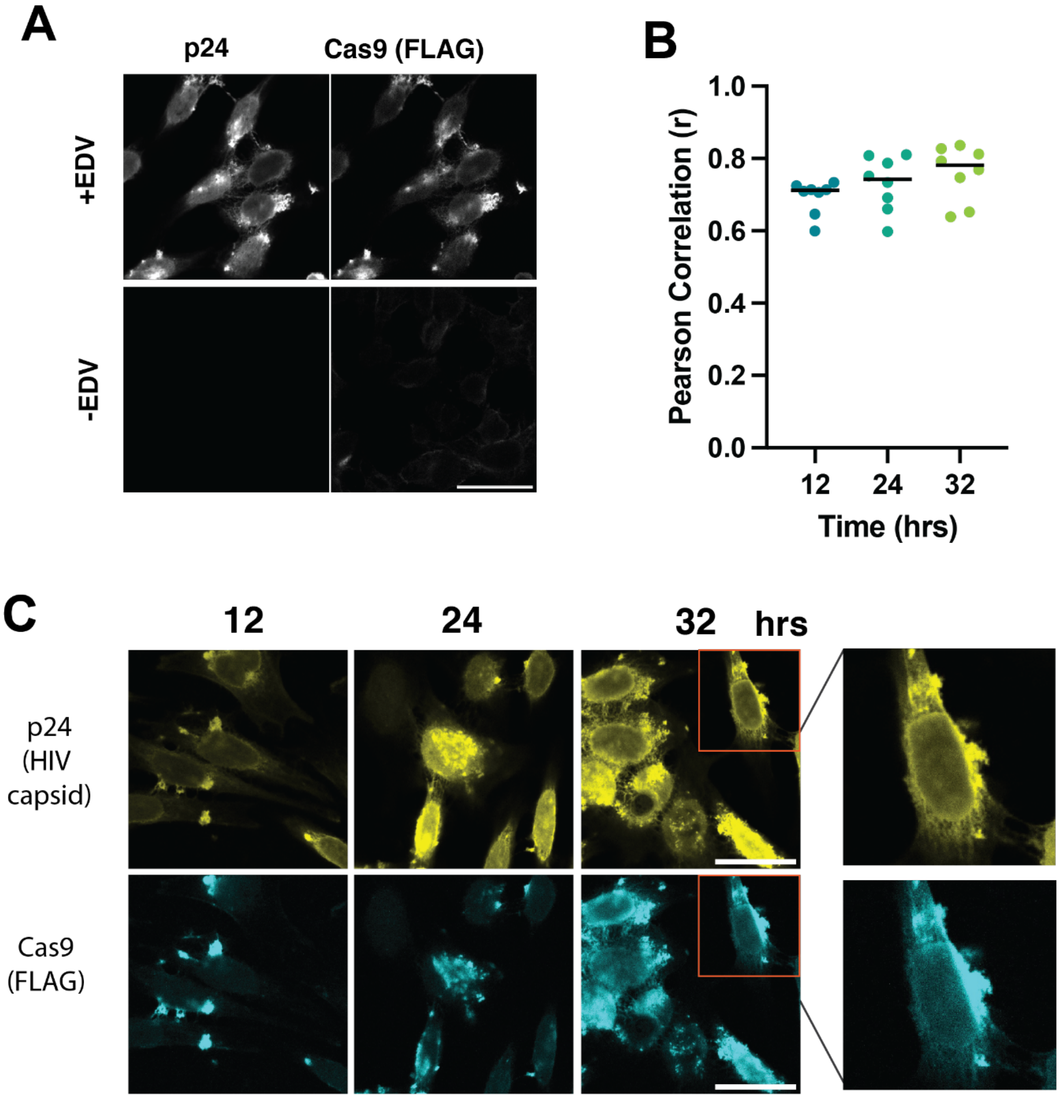

Supplemental Figure 10: Cas9 in EDV remains uncleaved from Gag, HIV structural polyprotein, in cells

(A) Validation of detection specificity for Cas9 and p24, a HIV capsid protein found in EDVs, stained in cells incubated with EDVs (350 ul 20x ultracentrifuged EDV, 1:1 dilution in OptiMEM) or OptiMEM media for 4 hours, then grown in supplemented DMEM until harvest at 32 hours (see Methods for details). Both EDV treated and untreated cells were stained with both primary and secondary antibodies (Supplemental Table 3). **(B)** Quantification of the colocalization of p24, a structural part of gag used in EDV packaging, and Cas9 at 12, 24 and 32 hours. Each data point represents an independent z-stack (n=8) quantified with Pearson's correlation analysis (HuygensPro, see Methods for details). **(C)** Representative images of EDV treated cells at 12, 24 and 32 hours stained to image p24 (Above) and Cas9 (Below), showing consistent colocalization of Cas9 and Gag.

Supplementary Table 1: gRNA and oligo sequences.

Guide RNA sequences and cloning sequences.

Protospacer	Gene target	fwd oligo	rev oligo	Source publication	NGS fwd	NGS rev
GAGTAGCGCGAGCACAGCTA	B2M-1	caccGAGTAGCGCGAGCACAGCTA	aaacTAGCTGTGCTCGCGCTACTC	10.1016/j.celrep.2021.109207	GCTCTTCCGATCTaagctgacagcattcgggc	GCTCTTCCGATCTgaagtcacggagcgagagag
GAGTCCGAGCAGAAGAAGAA	EMX1	caccGAGTCCGAGCAGAAGAAGAA	aaacTTCTTCTTCTGCTCGGACTC	10.1038/nbt.4066	GCTCTTCCGATCTCCGGAGGACAAAGTACAAACGGC	GCTCTTCCGATCTAAGCAGCACTCTGCCCTCGTG
GGTGAGTGAGTGTGTGCGTG	VEGFA3	caccGGTGAGTGAGTGTGTGCGTG	aaacCACGCACACACTCACTCACC	10.1038/nbt.4066	GCTCTTCCGATCTCTGGGTGAATGGAGCGAGCAG	GCTCTTCCGATCTGGAAGGCGGAGAGCCGGACA
CTTCAAGAGCAACAGTGCTG	TRAC3	caccCTTCAAGAGCAACAGTGCTG	aaacCAGCACTGTTGCTCTTGAAG	10.1016/j.cell.2023.08.041	GCTCTTCCGATCTTGCCTATTCACCGATTTT	GCTCTTCCGATCTATGCAAAGTCAGATTTGTTG
GGTACCTATCGATTGTCAGG	CCR5sp11	caccGGTACCTATCGATTGTCAGG	aaacCCTGACAATCGATAGGTACC	10.1038/nbt.4066	GCTCTTCCGATCTCTTCTGGGCTCACTATGCTGC	GCTCTTCCGATCTGGTGACCGTCCTGGCTTTTA

Additional B2M sequences. B2M-1 used as default unless otherwise stated.

**Protospacer**	**Target**	**Fwd oligo**	**Rev oligo**
GAGTAGCGCGAGCACAGCTA	B2M-1	caccGAGTAGCGCGAGCACAGCTA	aaacTAGCTGTGCTCGCGCTACTC
ACTCTCTCTTTCTGGCCTGG	B2M-2	caccACTCTCTCTTTCTGGCCTGG	aaacCCAGGCCAGAAAGAGAGAGT
gCTCGCGCTACTCTCTCTTTC	B2M-3	caccgCTCGCGCTACTCTCTCTTTC	aaacGAAAGAGAGAGTAGCGCGAGc
GGCCACGGAGCGAGACATCT	B2M-4	caccGGCCACGGAGCGAGACATCT	aaacAGATGTCTCGCTCCGTGGCC
AGGGTAGGAGAGACTCACGC	B2M-5	caccAGGGTAGGAGAGACTCACGC	aaacGCGTGAGTCTCTCCTACCCT
ACTCACGCTGGATAGCCTCC	B2M-6	caccACTCACGCTGGATAGCCTCC	aaacGGAGGCTATCCAGCGTGAGT
CGCGAGCACAGCTAAGGCCA	B2M-7	caccCGCGAGCACAGCTAAGGCCA	aaacTGGCCTTAGCTGTGCTCGCG

GGCCGAGATGTCTCGC TCCG	B2M-8	caccGGCCGAGATGTCTCGC TCCG	aaacCGGAGCGAGACATCTCGGC C
GCTACTCTCTCTTTCTG GCC	B2M-9	caccGCTACTCTCTCTTTCTG GCC	aaacGGCCAGAAAGAGAGAGTAG C

Anneal dsDNA substrates used for in vitro cleavage assay.

**Protospacer**	**Gene target**	**Fwd oligo**	**Rev oligo**
GAGTAGCGCGAGC ACAGCTA	B2M	/5Cy3/gcgtgcatgacattagcgag tagcgcgagcacagctaaggtcgta ctcgc	gcgagtacgaccttagctgtgctcgcgctactcgctaatg tcatgcacgc
GGTACCTATCGATT GTCAGG	CCR5	/5Cy5/gcgagtacgaCCTCCTGA CAATCGATAGGTACCgctaat gtcatgcacgc	gcgtgcatgacattagcGGTACCTATCGATT GTCAGGAGGtcgtactcgc
CTTCAAGAGCAAC AGTGCTG	TRAC3	/5Cy5/gcgagtacgaCCACAGCA CTGTTGCTCTTGAAGgctaat gtcatgcacgc	gcgtgcatgacattagcCTTCAAGAGCAAC AGTGCTGTGGtcgtactcgc

Guide sequence and NGS primers used for targeted deep sequencing specificity analysis.

**Protospacer**	**Target**	**Fwd primer**	**Rev Primer**
GAGTCCGAGCAG AAGAAGAA	EMX1	GCTCTTCCGATCTCCGGAGG ACAAAGTACAAACGGC	GCTCTTCCGATCTAAGCAGCACTCTG CCCTCGTG
	OT1	GCTCTTCCGATCTCTTTTATA CCATCTTGGGGTTACAG	GCTCTTCCGATCTCTAGGAAAGATTA ACAGAGAGTCTGAC
	OT2	GCTCTTCCGATCTCAATGTG CTTCAACCCATCACGGC	GCTCTTCCGATCTCCTCTACTTCATTG TACTCAAGGTAAG
GGTGAGTGAGTG TGTGCGTG	VEGFA3	GCTCTTCCGATCTCTGGGTG AATGGAGCGAGCAG	GCTCTTCCGATCTGGAAGGCGGAGA GCCGGACA
	OT1	GCTCTTCCGATCTGAAGGG GAGGGGGAAGTCACC	GCTCTTCCGATCTCGTGCGTGCCGCC GTTGATC
	OT2	GCTCTTCCGATCTTCTGTCA CCACACAGTTACCACC	GCTCTTCCGATCTGTAGTTGCCTGGG GATGGGGTATG
	OT3	GCTCTTCCGATCTCACCTGG CCCATTTCTCCTTTGAGG	GCTCTTCCGATCTTGGGGACAGCATG TGCAAGCCACA

Supplementary Table 2: Summary Values for in cellula FCS Experiments

For all fluorescence correlation spectroscopy (FCS) measurements, *n-BR* refers to the number of biological replicates and *n-Cells* refers to the number of individual cell measurements that passed all final filtering criteria across all biological replicates. The "FCS Mean" corresponds to the average nuclear concentration in nM measured for that condition. The "FCS SEM" is the standard error of the mean for the corresponding nuclear concentration measurement. Unless otherwise specified, the dosage is reported in the number of cas9 molecules per cell ($x10^7$) and in picomoles of Cas9 ribonucleoprotein complex per 10^5 cells. All of these experiments were done with B2M-1 (Supplementary Table 1) unless otherwise specified.

**Cell Type**	**Time (hrs)**	**Dosage (Cas9 x10^7 per cell)**	**Dosage (pmol/10^5 cells)**	***n* - BR**	***n* - FCS**	**FCS Mean**	**FCS SEM**
HeLa	12	7.5	12.5	2	28	22.1	1.10
HeLa	12	7.5	1.56	2	30	4.41	0.25
HeLa	24	15	25	2	33	38.6	2.58
HeLa	24	7.5	12.5	2	25	25.4	1.59
HeLa	24	7.5 (RNA only)	12.5 (RNA only)	2	26	19.4	0.84
HeLa	24	7.5 (B2M-9)	12.5 (B2M-9)	2	41	28.11	1.97
HeLa	24	3.8	6.25	3	37	8.11	0.65
HeLa	24	1.9	3.125	2	28	3.98	0.24
HeLa	24	0.94	1.56	2	37	4.22	0.24
HeLa	24	0.24	0.4	2	34	2.94	0.29
HeLa	36	7.5	12.5	2	40	25.0	1.61
HeLa	36	0.94	1.56	2	42	4.12	0.19
HeLa	48	7.5	12.5	2	50	5.58	0.34
HeLa	72	7.5	12.5	2	36	3.91	0.20
U2OS	24	7.5	12.5	2	26	21.4	1.96
U2OS	24	0.94	1.56	2	21	6.86	2.10
HEK293T	24	7.5	12.5	2	20	30.0	1.98

HEK293T	24	0.94	1.56	2	20	6.28	0.52

Supplementary Table 3: Antibodies used for Western Blots and Immunofluorescence microscopy

Antibodies Used:
WB= Western Blot; IF = immunofluorescence; mAb = monoclonal Antibody; pAb = polyclonal antibody

Primary Antibodies:

Antibody target/strain:	Source/Isotype:	Dilution(s) used:	Figures:	Company and Catalog #
CRISPR-Cas9	Rabbit pAb	1:1000 (WB), 1:100 (IF)	Fig. 4B (IF)	Diagenode (#C15310258)
FLAG (DDDDK sequence)	Rabbit Mab (recombinant)	1:250 (IF), 1:2500 (WB)	Fig. 4C (IF), Fig. S6D (WB), Fig. S10A,C (IF)	Abcam (#ab205606)
HIV1 p24	Mouse Mab	1:250 (IF), 1:1000 (WB)	Fig. S6D (WB), Fig. S10A,C (IF)	Invitrogen (#MA1-71515)
LAMP1	Rabbit Mab	1:100 (IF)	Fig. S9B,C (IF)	Cell Signaling Technology (#9091)
Rab5a	Mouse Mab	1:100(IF)	Fig. S9D,E (IF)	Invitrogen (#14-9711-82)

Secondary Antibodies:

Antibody:	Dilution(s) used:	Figures:	Company and Catalog #
IRDye® 680RD Goat anti-Mouse IgG Secondary Antibody	1:2500 (WB)	Fig. S6D	Licor (Catalog # 926-68070)
IRDye® 800CW Goat anti-Rabbit IgG Secondary Antibody	1:2500 (WB)	Fig. S6D	Licor (Catalog # 926-32211)
Goat anti-Rabbit IgG (H+L) Highly Cross-Adsorbed Secondary Antibody, Alexa Fluor™ Plus 405	1:1000 (IF)	Fig. 4B	Invitrogen (Catalog # A48254)

Goat anti-Rabbit IgG (H+L) Highly Cross-Adsorbed Secondary Antibody, Alexa Fluor™ Plus 555	1:1000 (IF)	Fig. S9B,D	Invitrogen (Catalog # A32723)
Alexa Fluor™ 594 Tyramide SuperBoost™ Kit, goat anti-rabbit IgG	Used in accordance with manufacturer's instructions.	Fig. 4D, Fig. S10A,C	ThermoFisher (Catalog # B40944)
Alexa Fluor™ 488 Tyramide SuperBoost™ Kit, goat anti-mouse IgG	Used in accordance with manufacturer's instructions.	Fig. S10A,C	ThermoFisher (Catalog # B40941)

Counterstain:	Cellular Compartment:	Dilution(s) used:	Figures:	Company and Catalog #
SYTOX™ Deep Red Nucleic Acid Stain	Nucleus	1:2000 (IF)	Fig. 4B,C	Invitrogen (#S11380)

References

1. Sternberg,S.H., Redding,S., Jinek,M., Greene,E.C. and Doudna,J.A. (2014) DNA interrogation by the CRISPR RNA-guided endonuclease Cas9. *Nature*, **507**, 62–67.

2. Gong,S., Yu,H.H., Johnson,K.A. and Taylor,D.W. (2018) DNA Unwinding Is the Primary Determinant of CRISPR-Cas9 Activity. *Cell Rep.*, **22**, 359–371.

3. Casini,A., Olivieri,M., Petris,G., Montagna,C., Reginato,G., Maule,G., Lorenzin,F., Prandi,D., Romanel,A., Demichelis,F., *et al.* (2018) A highly specific SpCas9 variant is identified by in vivo screening in yeast. *Nat. Biotechnol.*, **36**, 265–271.
